# Metabolomic Profiling of Extracellular Vesicles Reveals Distinct Metabolic Dysregulation and Treatment-Specific Signatures in Depression

**DOI:** 10.3390/biom16040533

**Published:** 2026-04-02

**Authors:** Nikola Balic, Gordana Nedic Erjavec, Marcela Konjevod, Jorge Saiz, Tina Curkovic, Lucija Tudor, Dubravka Svob Strac, Alja Videtic Paska, Julija Smon, Magda Tusek-Znidaric, Marina Sagud, Bjanka Vuksan Cusa, Tea Fabijanic, Zrinka Pesut, Biljana Kosanovic Rajacic, Zoran Bradas, Nela Pivac, Matea Nikolac Perkovic

**Affiliations:** 1 Division of Molecular Medicine, Ruđer Bošković Institute, 10000 Zagreb, Croatia; nbalic@irb.hr (N.B.); gnedic@irb.hr (G.N.E.); mkonjev@irb.hr (M.K.); tmilos@irb.hr (T.C.); lucija.tudor@gmail.com (L.T.); dsvob@irb.hr (D.S.S.); npivac@irb.hr (N.P.); 2 Department of Food Science and Technology, College of Agriculture and Environmental Sciences, University of California, Davis, CA 95616, USA; jorge.saiz@bcmaterials.net; 3 BCMaterials, Basque Center for Materials, Applications and Nanostructures, UPV/EHU Science Park, 48940 Leioa, Spain; 4 Institute for Biochemistry and Molecular Genetics, Faculty of Medicine, University of Ljubljana, 1000 Ljubljana, Slovenia; alja.videtic@mf.uni-lj.si (A.V.P.); julija.smon@mf.uni-lj.si (J.S.); 5 National Institute of Biology Ljubljana, 1000 Ljubljana, Slovenia; magda.tusek.znidaric@nib.si; 6 Department for Psychiatry and Psychological Medicine, University Hospital Center Zagreb, 10000 Zagreb, Croatia; marinasagud@mail.com (M.S.); bjanka.vuksan@gmail.com (B.V.C.); bkosanov@gmail.com (B.K.R.); zbradas@gmail.com (Z.B.); 7 Department for Emergency Medicine, University Hospital Center Zagreb, 10000 Zagreb, Croatia; tea.fabijan@gmail.com; 8 Vuk Vrhovac University Clinic for Diabetes, Endocrinology and Metabolic Diseases, Merkur University Hospital, 10000 Zagreb, Croatia; zrinka.vuksan@gmail.com; 9 University Psychiatric Hospital Vrapče, 10000 Zagreb, Croatia

**Keywords:** MDD, TRD, metabolomics, extracellular vesicles, duloxetine, bright-light therapy, esketamine, treatment

## Abstract

Major depressive disorder (MDD) is associated with complex metabolic alterations. In this study, we applied a multiplatform metabolomics approach (GC-MS and LC-MS) to characterize the plasma extracellular vesicle (EV) metabolome in healthy controls (N = 50), responsive MDD patients (N = 60), and patients with treatment-resistant depression (TRD; N = 65). Longitudinal analyses were performed following 8-week treatment with duloxetine (N = 30), bright-light therapy (BLT; N = 30), or esketamine (N = 35). A total of 230 metabolites were identified, with the most pronounced metabolic alterations observed in TRD patients, particularly in lipid, amino acid, and energy metabolism pathways. Elevated lysophospholipids and fatty acids in TRD suggested dysregulated lipid metabolism and inflammatory processes. All treatments resulted in clinical improvement, accompanied by partial normalization of metabolic profiles. Duloxetine treatment was associated with modulation of amino acid and glycerophospholipid metabolism, including increases in tryptophan-related metabolites and normalization of specific lipid species. BLT primarily reduced lysophospholipids and mannose levels, while esketamine modulated metabolites related to lipid turnover, short-chain fatty acids, carbohydrate metabolism, and neuroendocrine function, including increased thyrotropin-releasing hormone levels. These findings support the concept that TRD represents a biologically distinct and more metabolically dysregulated subtype of depression and highlight EV-based metabolomics as a promising approach for elucidating disease and treatment mechanisms.

## 1. Introduction

Major depressive disorder (MDD) is a heterogeneous and highly prevalent mood disorder, representing one of the leading global public health challenges [1]. The etiology of MDD is multifactorial and reflects complex interactions among genetic susceptibility, developmental and environmental influences, and neurobiological and metabolic processes [2]. Pharmacological treatment most commonly includes selective serotonin reuptake inhibitors (SSRIs), serotonin–norepinephrine reuptake inhibitors (SNRIs), tricyclic and tetracyclic antidepressants, and monoamine oxidase inhibitors. Among these agents, duloxetine is a second-generation SNRI approved for the treatment of MDD since 2004. Duloxetine exerts its therapeutic effects primarily through inhibition of serotonin and norepinephrine transporters, thereby increasing the synaptic availability of these monoamines [3]. In addition, it increases dopamine concentrations in the prefrontal cortex [3]. While monoaminergic agents such as duloxetine have long represented the pharmacological cornerstone of treatment, the need for faster and more robust therapeutic responses has prompted the development and integration of additional treatment modalities, including BLT and esketamine. In recent years, rapid-acting antidepressant therapies have emerged as important alternatives for patients with treatment-resistant depression (TRD). Among these, esketamine, the S-enantiomer of ketamine, has been approved as an intranasal treatment for adults with TRD. Esketamine exerts its primary pharmacological effect through noncompetitive antagonism of N-methyl-D-aspartate (NMDA) receptors, leading to increased glutamatergic transmission and activation of neuroplasticity-related signaling pathways [4]. The precise biological mechanisms underlying esketamine’s rapid and sustained antidepressant effects remain incompletely understood, and reliable biomarkers capable of predicting treatment response are currently lacking. Beyond pharmacotherapy, several adjunctive therapeutic strategies may be incorporated into the treatment of MDD. One such approach is bright-light therapy (BLT), which has long been established as a first-line treatment for seasonal affective disorder and has increasingly demonstrated efficacy in non-seasonal depression when used as an adjunct to pharmacological treatment [5]. Despite their differing primary targets, accumulating evidence suggests that these interventions may converge on shared downstream mechanisms, particularly the enhancement of neuroplasticity and the upregulation of brain-derived neurotrophic factor [6]. However, important gaps remain in our understanding of how these mechanisms interact when such treatments are used in combination. Elucidating these potential therapeutic synergies is essential not only for optimizing treatment strategies in patients with TRD, but also for identifying biological markers associated with treatment response across these distinct yet interconnected neurobiological pathways.

In this context, increasing attention has been directed toward the identification of biological signatures that may capture the molecular processes underlying differential treatment outcomes. One promising approach involves metabolomics, which enables comprehensive profiling of small-molecule metabolites in biological samples and provides a functional readout of ongoing biochemical processes. Accumulating evidence indicates that several metabolic pathways are implicated in the pathophysiology of depression, particularly those related to tryptophan metabolism and the kynurenine pathway [7]. Disturbances in cellular energy metabolism, including mitochondrial dysfunction and alterations in central carbon metabolism, have also been increasingly reported in patients with MDD [8]. However, despite growing evidence implicating metabolic dysregulation in depression [9], the metabolic signatures associated with differential responses to emerging multimodal treatment strategies remain poorly characterized. Extracellular vesicles (EVs) represent a heterogeneous class of membrane-bound particles secreted by nearly all cell types and present in diverse biofluids. Functioning as essential mediators of intercellular communication, EVs transport a complex molecular cargo, including proteins, lipids, and small-molecule metabolites, that reflects the physiological state of their cell of origin [10]. EVs isolated from peripheral blood have emerged as a “liquid biopsy” for the central nervous system. Because EVs, particularly those of endosomal origin, are capable of crossing the blood–brain barrier, they carry molecular signatures derived from brain tissue into the systemic circulation [11]. Consequently, the metabolomic profiling of circulating EVs offers a unique window into the neuro-metabolic dysregulation of MDD and the biochemical mechanisms of antidepressant response [12]. Therefore, this study aimed to investigate the metabolomic profiles of plasma EVs associated with differential treatment responses in patients with responsive MDD and TRD, using a multiplatform metabolomics approach (gas chromatography (GC) and liquid chromatography (LC) coupled with mass spectrometry (MS)). Additionally, we aimed to identify metabolite signatures that distinguish healthy controls from both MDD cohorts at baseline. By utilizing an untargeted multiplatform approach, we sought to delineate the longitudinal biochemical shifts induced by three distinct therapeutic modalities: duloxetine pharmacotherapy, adjunctive BLT, and intranasal esketamine. Through this high-resolution profiling of EV cargo, we aimed to provide deeper insights into the multisystem pathophysiology of antidepressant response.

## 2. Materials and Methods

### 2.1. Study Population and Clinical Design

This prospective clinical study enrolled a total of 125 patients diagnosed with MDD according to the criteria of the Diagnostic and Statistical Manual of Mental Disorders, Fifth Edition, Text Revision (DSM-5-TR) [13], and 50 healthy control subjects at the University Hospital Centre Zagreb, Department of Psychiatry. The MDD cohort was divided into two distinct groups based on treatment history, partial responders (N = 60) and patients with TRD (N = 65). Partial responders included patients currently treated with an SSRI or SNRI showing a partial therapeutic response, defined as a 25–49% reduction in the Hamilton Depression Rating Scale (HAMD-17) and Montgomery–Åsberg Depression Rating Scale (MADRS) total scores. These patients were assigned to either duloxetine or adjunctive BLT by the treating clinician, without consideration of age, sex, or other factors not relevant to clinical decision-making. Patients in the TRD group had a history of at least two failed antidepressant trials of adequate dose and duration. This group received adjunctive intranasal esketamine in addition to their ongoing SSRI/SNRI regimen. In addition, a healthy control group (N = 50) was included, consisting of volunteers with no history of psychiatric disorders, and/or psychiatric medication use, comparable to the patient cohort by age and sex, to ensure demographic comparability.

#### 2.1.1. Inclusion and Exclusion Criteria

General inclusion criteria for all participants were: age 18–70 years, both sexes, and provided written informed consent. Exclusion criteria across all groups included: use of tryptophan or St. John’s wort within the previous 3 months, current therapy with antipsychotics or opiates, presence of psychotic symptoms, substance or alcohol use disorders, epilepsy, eating or neurodegenerative disorders, intellectual disabilities, and severe somatic illnesses (e.g., uncontrolled hypertension, diabetes, or thyroid disease). Additionally, individuals who were pregnant, breastfeeding, following restrictive diets, or using weight-loss pharmacotherapy were excluded. Active psychotherapy was an exclusion criterion, with the exception of standardized supportive psychotherapy. Supportive psychotherapy includes collecting new information regarding the psychological symptoms, environmental circumstances, and potential adverse effects of drugs, along with empathetic listening and providing advice and education. On the contrary, active psychotherapy includes, but is not restricted to, cognitive behavioral therapy, any type of psychodynamic-oriented psychotherapy, exposure therapy, family or couples psychotherapy, group psychotherapy, and interpersonal psychotherapy. For the partial-responder group, additional exclusion criteria included a prior diagnosis of TRD or a previous lack of response/current treatment with duloxetine or BLT. For TRD patients, additional exclusion criteria were: known aneurysmal vascular disease, history of intracerebral hemorrhage, and recent (within 6 weeks) cardiovascular event. For BLT, additional exclusion criteria were: the presence of eye disease such as glaucoma, retinopathies, and eye infection.

#### 2.1.2. Treatment Protocols and Assessment

Duloxetine was administered at 60–120 mg daily with standard clinical titration. BLT was administered in the morning, three times weekly for eight weeks using 10,000 lux white fluorescent light. Esketamine was self-administered via nasal spray under clinical supervision. In the first four weeks, esketamine was administered twice weekly, and in the following four weeks, the frequency was reduced to once weekly. Esketamine doses were 28 to 84 mg per application. Clinical assessments (HAMD-17 and MADRS) and biological sampling were conducted at baseline and after eight weeks of treatment for a longitudinal analysis of clinical and metabolic outcomes. Healthy controls underwent a single baseline assessment and sampling following identical pre-analytical procedures.

#### 2.1.3. Final Analytical Cohort

For the baseline comparison between healthy controls, MDD patients (subsequently randomized to duloxetine or BLT), and TRD patients, all participants who fulfilled the predefined inclusion and exclusion criteria were included (total N = 175). For the assessment of specific treatment effects on the metabolic profile, the analysis was restricted to those participants who fulfilled all inclusion criteria and demonstrated a partial therapeutic response by the end of the 8-week period (total N = 94). In the partial-responder group, all 60 participants met this threshold. In the TRD group, consistent with the known clinical profile of the drug, a higher attrition rate was observed due to dropouts and non-response (defined as <25% improvement on HAMD-17/MADRS). Consequently, the final assessment of the metabolic profile for the esketamine intervention was conducted on a cohort of 34 patients. A simplified schematic representation of the study design is presented in Figure S1 (Supplementary Materials).

### 2.2. Blood Sample Collection

Venous blood samples were collected between 7:00 and 9:00 a.m., following an overnight fast, integrated into routine laboratory visits. Samples were drawn into tubes containing EDTA as an anticoagulant (BD Vacutainer™ tubes, Becton, Dickinson and Company, Franklin Lakes, NJ, USA). Immediately following collection, platelet-poor plasma was isolated through a series of centrifugation steps (3 min at 1100× *g*, followed by 15 min at 5030× *g*). Plasma aliquots (1 mL) for subsequent EV isolation were immediately frozen and stored at –80 °C until further analysis.

### 2.3. EV Isolation by Ultracentrifugation

For the isolation of EVs and subsequent metabolite extraction, 1 mL plasma aliquots were utilized. Samples were first centrifuged at 10,000× *g* for 20 min at 4 °C to eliminate cellular debris, large protein aggregates, and lipoprotein complexes. A 900 µL volume of the resulting supernatant was diluted in 8 mL of Dulbecco’s phosphate-buffered saline (dPBS) (Sigma-Aldrich, Steinheim, Germany), gently homogenized, and transferred into 11 mL polypropylene ultracentrifuge tubes. A sucrose gradient was prepared by layering the plasma–PBS mixture over 2 mL of 20% sucrose in dPBS. The samples were then subjected to ultracentrifugation (Optima MAX-XP ultracentrifuge with MLA-55 rotor, Beckman Coulter, Brea, CA, USA) at 100,000× *g* for 2 h and 15 min at 4 °C. After centrifugation, for each sample, the supernatant was carefully aspirated, and the resulting EV-enriched pellet was resuspended in 30 µL of dPBS and stored at –80 °C until further analysis.

### 2.4. Validation and Confirmation of Isolated EVs

Following the Minimal Information for Studies of Extracellular Vesicles 2018 (MISEV2018) guidelines, EVs were considered a mixed population of small (<200 nm) and medium/large (>200 nm) vesicles. EV characterization was performed using Western blot, nanoparticle tracking analysis (NTA), and transmission electron microscopy (TEM) (representative figures are presented as Figure S2, Supplementary Materials). Western blot analysis was performed as previously described [14]. Primary antibodies used against EVs markers were: flotillin-1 (Cell Signaling Technology, Beverly, MA, USA), HSP70 (Santa Cruz, Dallas, TX, USA), CD9 (Santa Cruz, Dallas, TX, USA), calnexin (Sigma Aldrich, St. Louis, MO, USA), TSG101 (Sigma Aldrich, St. Louis, MO, USA), and cytochrome-c (Cyc-c; Sigma Aldrich, St. Louis, MO, USA). Western blotting was used for qualitative validation of extracellular vesicle markers only. EV size distribution and concentration were measured using a NanoSight N300 (Malvern Panalytical, Malvern, UK). NTA was performed as previously described [14]. For TEM, 4 µL of uEV samples were adsorbed onto glow-discharged formvar-carbon copper grids, stained with 1% uranyl acetate, and examined using a TALOS L120 transmission electron microscope (100 kV) (Thermo Fisher Scientific, Waltham, MA, USA). At least 10 grid squares were inspected, and vesicle size was measured from representative micrographs (N = 100 per sample) using Velox v3.0 software (Thermo Fisher Scientific, Waltham, MA, USA).

### 2.5. Metabolite Extraction from Plasma EVs

Samples were thawed on ice and gently mixed. They were then incubated on ice for 30 s, followed by 90 s at 37 °C. Samples were ultrasonicated for 60 s, until the solution became clear. Cold H_2_O (110 µL) was added to each sample and vortex-mixed thoroughly. For metabolite extraction, a mix of methanol (LC-MS grade, Sigma-Aldrich, Steinheim, Germany), acetonitrile (ACN, LC-MS grade, Sigma-Aldrich, Steinheim, Germany) and methyl tert-butyl ether (MTBE, Sigma-Aldrich, Steinheim, Germany) was used. A total of 360 µL of ice-cold MeOH:ACN:MTBE extraction mixture (1:1:1, *v*/*v*/*v*) was added. Samples were vortex-mixed for 60 s, ultrasonicated for 60 s, vortex-mixed again for 60 s, and incubated at −20 °C for 1 h. After incubation, the samples were centrifuged (22,600× *g* for 15 min at 4 °C). Afterwards, 150 µL was transferred to GC-MS vials and 300 µL to LC-MS vials. Additionally, 15 µL from each sample was pooled to generate quality control (QC) samples. The samples were evaporated to dryness using an Eppendorf^®^ Concentrator Plus (Eppendorf, Hamburg, Germany). Dried extracts were stored at −80 °C until the analysis by LC-MS and GC-MS.

### 2.6. Preparation of Samples for Metabolomics Analyses

For GC-MS analysis, dried extracts were derivatized before measurement. Methoximation was performed by adding 10 μL of O-methoxyamine hydrochloride (15 mg/mL in pyridine; Sigma-Aldrich, Steinheim, Germany). Samples were vortexed for 5 min, followed by three cycles of ultrasonication (2 min) and vortex mixing (2 min), and then incubated in the dark at room temperature for 16 h. For silylation, 10 μL of N,O-bis(trimethylsilyl)trifluoroacetamide with 1% trimethylchlorosilane (BSTFA + 1% TMCS; Pierce Chemical Co., Rockford, IL, USA) was added. Samples were vortexed for 5 min and incubated at 70 °C for 1 h. After cooling to room temperature, 100 μL of methyl stearate (20 ppm in heptane; Sigma-Aldrich, Steinheim, Germany) was added as an internal standard, followed by vortex mixing for 2 min. For LC–MS analysis, EV pellets were reconstituted in cold methanol (LC-MS grade, Sigma-Aldrich, Steinheim, Germany) and vortex-mixed thoroughly before analysis. Blank samples were prepared using the same protocol as EV samples, starting from the metabolite extraction step. Quality control (QC) samples were processed identically.

### 2.7. GC-MS Analysis

Metabolomic fingerprinting of EV samples was performed using an Agilent 7890A GC equipped with an Agilent 7693 autosampler and coupled to an inert quadrupole MS (Agilent 5975; Agilent Technologies, Santa Clara, CA, USA). A volume of 2 μL of each sample was injected in split mode (1:10) into a Restek 20782 deactivated glass-wool split liner (Bellefonte, PA, USA). Chromatographic separation was achieved on a DB-5MS column (30 m × 0.25 mm × 0.25 μm; 95% dimethylpolysiloxane/5% diphenylpolysiloxane) with a 10 m pre-column (J&W integrated with Agilent 122-5532G). Helium was used as the carrier gas at a constant flow rate of 1 mL/min. The injector temperature was set to 250 °C. The oven temperature program started at 60 °C (1 min hold), increased at 10 °C/min to 325 °C, and was held for 10 min. The transfer line temperature was 290 °C, while the ion source and quadrupole temperatures were set to 230 °C and 150 °C, respectively. The total run time was 37.5 min per sample. Mass spectra were acquired in electron ionization (EI) mode at 70 eV, scanning a mass range of *m*/*z* 50–600 at a rate of 2 spectra/s.

### 2.8. LC-MS Analysis

The samples were analyzed in an Agilent 1290 Infinity II coupled to a Revident G6575A LC-Q-TOF MS system (Agilent Technologies, Santa Clara, CA, USA). The mobile phase A was 0.1% (*v*/*v*) formic acid (MS grade, Sigma-Aldrich, Steinheim, Germany) in water, and the mobile phase B was 0.1% (*v*/*v*) of formic acid (MS grade, Sigma-Aldrich, Steinheim, Germany) in ACN (LC-MS grade, Sigma-Aldrich, Steinheim, Germany). The injection volume was set to 2 µL, using a multi-wash of the injection needle and port with the initial chromatographic conditions. The temperature in the oven was maintained at 30 °C during the whole analysis, which was carried out at a flow rate of 0.250 mL/min. The chromatographic run started at 0% B, which was maintained for 1.00 min. Then, it was linearly increased up to 100% B at minute 10.00. These conditions were maintained until min 12.00, and then the initial conditions were recovered in min 12.01 and maintained for the column re-equilibration until min 15.00. A dual AJS ESI was used in positive ionization mode and negative ionization modes in separate analyses. For positive and negative ionization modes, the gas temperature was set at 250 °C, the drying gas flow at 8 L/min, the nebulizer pressure at 30 psi, the sheath gas temperature at 350 °C, and the sheath gas flow at 11 L/min. In positive mode, the capillary voltage was 3500 V, the nozzle voltage was set to 500 V, and the fragmentor voltage was 150 V. In negative mode, the capillary voltage was 3000 V, the nozzle voltage was set to 0 V, and the fragmentor voltage was 150 V. The Oct 1 RF Vpp was 750 V, and the skimmer was configured to 45 V. For both polarities, the system was operated in auto MS/MS mode. For the data recording in centroid mode for the MS-only acquisition, the range was set from 20 to 1200 *m*/*z* at 4 spectra/s. The MS/MS acquisition was done between 20 and 1200 *m*/*z*, also at 12 spectra/s. Three maximum precursor ions per cycle were selected, with a narrow iso. window and an absolute threshold of 1000 counts. The reference masses used during the analyses were 119.03632 and 966.00073.

### 2.9. Data Treatment

#### 2.9.1. GC-MS Data Treatment

The quality of total ion chromatograms (TICs) for all samples, quality controls (QCs), and blanks was evaluated using Agilent MassHunter Qualitative Analysis software (version 10.0). Reproducibility of the internal standard was verified in all samples. Raw data files were imported into Agilent MassHunter Unknowns Analysis (version 10.0) for deconvolution and compound identification using targeted spectral libraries (Fiehn library v2013 and the in-house CEMBIO plasma spectral library). Compounds were identified based on retention time (RT) and mass spectra. Additional verification of identified compounds and unknown features was performed using the NIST library (National Institute of Standards and Technology, 2017 version). Data were aligned using Agilent MassProfiler Professional (version 13.0) and exported to MassHunter Quantitative Analysis (version B10.0) for peak integration. Compound abundances were normalized to the internal standard, and blank subtraction was performed before statistical analysis.

#### 2.9.2. LC-MS ESI Data Treatment

The quality of total ion chromatograms (TICs) for all samples, quality controls (QCs), and blanks was evaluated using Agilent MassHunter Qualitative Analysis software (version 10.0). Data-dependent acquisition (DDA) data was treated with MS-DIAL v5.3.240719 [15] for deconvolution, peak detection, and alignment. MS1 and MS2 tolerances were 0.010 and 0.025 Da, respectively. For the data collection, the retention time started at min 0.0 and ended in min 12.5. The minimum peak height for peak detection was 3000, and the mass slice was 0.1 Da. Smoothing based on a linear weighted moving average was applied to the data at a level of 3, with a minimum peak width of 5 scans. For the deconvolution, a sigma window value of 0.5 was applied, with an MS/MS abundance cut off of 0 in amplitude. Masses above the precursor ions were excluded, keeping the isotopic ions until 5 Da above them. For peak alignment, the retention time tolerance was 0.1 min and the MS1 alignment was set to 0.015 Da. The retention time factor and the MS1 factor for alignment were both set to 0.5. No peaks were excluded based on their count, and the blank subtraction was performed at a later stage.

### 2.10. Statistical Analysis

Before the statistical analysis, the raw data obtained by both GC-MS and LC-MS were filtered based on the criteria proposed by Godzien and colleagues [16]. Feature detection thresholds were predefined in order to distinguish true metabolite signals from noise in untargeted MS data. The variables were retained if they exceeded a signal-to-noise ratio of >5, were present in ≥80% of the QCs (with relative standard deviation (RSD) <30% in QC samples), or were present in <20% of the QCs but also present in ≥50% of the samples in a specific subject group. Also, only peaks with intensity > 1000 counts were kept to avoid very weak and unreliable signals. In order to correct for the possible intra-batch effect, we used the Quality Control-Robust Spline Correction (QC-RSC) algorithm, as suggested by Kuligowski and colleagues [17]. Support vector regression (QC-RSC) was performed using MATLAB (7.10.0.499, MathWorks, Natick, MA, USA) and the LIBSVM library [18]. After eliminating intra-batch effects, the data were normalized in order to decrease the unwanted variations that may result from the errors in the sample preparation [19]. Auto scaling (Unit Variance, UV) and log transformation were used to normalize and scale metabolic signals before statistical analyses when appropriate [20]. For GC-MS data, the abundance of detected compounds was additionally normalized by the signal of the internal standard in each sample. If necessary, the missing values in data sets were replaced using KNN (K-Nearest Neighbors) imputation.

For multivariate statistical analyses, the SIMCA-P+ software (version 15.0.2.5959, Umetrics, Umea, Sweden) and MetaboAnalyst 6.0 [21] were used. This includes building up Principal Component Analysis (PCA) models and Orthogonal Partial Least Squares Discriminant Analysis (OPLS-DA) models. Based on OPLS-DA models, variable importance in projection (VIP) values were obtained. Permutation analyses were performed for the obtained multivariate OPLS-DA models to assess their reliability. Possible overfitting of the OPLS-DA model was further evaluated by cross-validated ANOVA (CV ANOVA).

Univariate statistical analyses were done using MetaboAnalyst 6.0 [21] and MATLAB (7.10.0.499, MathWorks, Natick, MA, USA). The normal distribution was evaluated using the Kolmogorov–Smirnov test. The comparison of metabolite abundances between specific independent groups was done using Student’s *t*-test, Mann–Whitney U test, analysis of variance (ANOVA; with Tukey’s Honestly Significant Difference (HSD) post hoc comparisons test), or Kruskal–Wallis ANOVA by ranks (with Dunn’s multiple post hoc comparisons test), depending on data distribution and number of groups compared. The comparison of metabolite abundances between specific dependent groups was done using the Paired Samples *t*-test or Wilcoxon signed-rank test, depending on data distribution. All these analyses were followed by Benjamini–Hochberg (FDR, false discovery rate) post hoc correction for multiple comparisons. For the results obtained with the univariate statistical approach, we reported comparisons that resulted in *p*-value ≤ 0.05, and VIP > 1.0. However, we also pointed out the q-value (FDR-adjusted *p*-value) for all these comparisons. While in some cases the FDR-corrected q-values did not reach the threshold of significance, metabolites with a nominal *p*-value ≤ 0.05 and VIP > 1.0 were included in the results to ensure a comprehensive exploratory analysis. This approach was chosen to prioritize the identification of biologically relevant patterns and to provide a basis for future targeted validation studies, as strict multiplicity adjustments may lead to an unacceptable loss of potentially important biological information. The magnitude of metabolic shifts was expressed as fold change (FC) and log2FC. The FC value was calculated as the ratio of the mean metabolite abundance in the patient cohort to that of the healthy control group. When evaluating the effect of treatment, FC was calculated as the ratio of the mean metabolite abundance in the patient cohort after treatment to that of the same group of patients at baseline. Demographic and clinical characteristics of the participants deviated from the normal distribution (tested with the Kolmogorov–Smirnov test); therefore, the non-parametric Mann–Whitney U test and the Kruskal–Wallis ANOVA by ranks (with Dunn’s multiple post hoc comparisons test) were used to compare the subject groups. When comparing the frequency of male and female subjects between healthy controls and patient cohorts, a χ^2^-test was used.

The sample size was determined a priori using the tool G*Power 3.1.9.2 [22]. To achieve a power of 80%, with fixed α = 0.05 and effect size d = 0.25, when determining differences between three independent groups of subjects, the total sample size needed was 159. When comparing two independent groups, the total sample size needed was 102 (Student *t*-test) or 106 (Mann–Whitney U test), and for matched pairs, the needed sample size per group was 28 (Wilcoxon signed-rank test) or 27 (Paired Samples *t*-test) (power of 80%, with fixed α = 0.05 and effect size d = 0.5).

## 3. Results

### 3.1. Participants

The initial study population comprised of 175 participants across three cohorts: healthy controls: HC (N = 50), patients with MDD showing a partial response to SSRI/SNRI therapy (N = 60), and patients with TRD (N = 65). Comprehensive demographic and clinical characteristics are summarized in Table 1. Statistical analysis revealed no significant differences between the groups regarding sex distribution (*p* = 0.142) or age (*p* = 0.058), ensuring demographic comparability. A significant difference was observed in baseline HAMD-17 scores (*p* = 0.004), and MADRS scores (*p* < 0.001). This discrepancy was driven by higher HAMD-17 and MADRS total scores in the TRD group, reflecting a greater symptom burden at the time of enrollment (Table 1).

Of the 65 patients initially enrolled in the TRD cohort, 34 (52.3%) completed the 8-week esketamine protocol and met the clinical criteria for the final longitudinal metabolomics analysis. A significant portion of the TRD cohort experienced premature discontinuation, primarily driven by treatment intolerance related to the side-effect profile of intranasal esketamine (N = 13), due to an inadequate response to treatment (N = 10), or logistical non-adherence to the induction phase schedule (N = 8). To ensure that the metabolic analysis specifically captured the biochemical correlates of therapeutic improvement, participants who failed to achieve a partial therapeutic response (defined as a <25% reduction in HAMD-17 and MADRS scores) were excluded from the final assessment. In contrast, the partial-responder subgroups (duloxetine and BLT) demonstrated a 100% completion and response rate (N = 60). Consequently, the longitudinal assessment of treatment-specific metabolic effects was conducted on a final cohort of 94 participants: 30 receiving duloxetine, 30 receiving adjunctive BLT, and 34 receiving adjunctive intranasal esketamine. Detailed demographic and clinical comparisons for this final analytical subset are provided in Table 2.

The three analytical subgroups (duloxetine, BLT, and esketamine) were well-matched at baseline, with no significant differences in age (*p* = 0.792) or sex distribution (*p* = 0.170). However, the baseline HAMD-17 (*p* = 0.011) and MADRS (*p* < 0.001) scores differed significantly, reflecting the higher symptom severity inherent to the TRD population compared to those randomized to duloxetine or BLT (Table 1). Following the 8-week intervention period, this baseline difference was no longer detectable (*p* = 0.939), indicating a convergence of clinical status across all treatment groups.

### 3.2. Differential Profiling of EV-Derived Metabolites Across Healthy Controls, MDD, and TRD Patients

To achieve a comprehensive mapping of the plasma EV metabolome, we utilized a multiplatform analytical approach combining GC-MS and LC-M in both positive and negative ionization modes. Following rigorous data filtration, deconvolution, and curation to ensure high-confidence identifications, a total of 230 unique metabolites were retained for downstream statistical analysis. The GC-MS platform contributed 40 distinct compounds, primarily representing small organic acids and amino acids. The LC-MS analysis further expanded metabolic coverage, identifying 104 metabolites in positive ionization mode and 86 in negative ionization mode, predominantly comprising diverse lipid species and complex polar metabolites.

Multiplatform untargeted metabolomic analysis of plasma EVs revealed distinct metabolic signatures across patients with MDD, TRD, and healthy controls. To initially assess the variance in the global metabolomic profiles, an unsupervised PCA was performed (Figure S3, Supplementary Materials). While the PCA indicated overlapping metabolic spaces among the three cohorts, a modest separation was visible between the healthy controls and TRD groups. Subsequent ANOVA on log-transformed data or Kruskal–Wallis ANOVA on ranks (Table S1, Supplementary Materials) confirmed that the most pronounced metabolic shifts occurred in the TRD group when compared to both the healthy and the MDD cohort. In contrast, the baseline differences between healthy individuals and the MDD group were less marked, suggesting a more subtle metabolic perturbation in non-TRD patients. Across all comparisons, 34 metabolites were identified as significantly altered in relative abundance (Table S1, Supplementary Materials). These compounds represent diverse chemical classes, including benzene derivatives, organooxygen compounds, carboxylic acids, fatty acyls, glycerolipids, glycerophospholipids, and imidazopyrimidines. Notably, while ceramides and bis(monoacylglycero)phosphates (BMPs) were detected within the EV cargo, their abundance remained stable across all study groups, suggesting these specific lipid classes may not be primary drivers of the observed depressive phenotypes in this cohort.

To further resolve the metabolic differences between the cohorts, supervised OPLS-DA was employed (Figure S4, Supplementary Materials). Models were constructed for pairwise comparisons (healthy controls vs. MDD, healthy controls vs. TRD, and MDD vs. TRD). These models demonstrated a modest but consistent separation between the groups (Figure S4, Supplementary Materials). To identify the metabolites primarily responsible for this discrimination, VIP scores were calculated, with those <1.00 prioritized for further evaluation (Table 3). Permutation testing (200 iterations) was performed to validate all OPLS-DA models by randomly shuffling class labels, thereby assessing whether the observed predictive ability (Q^2^) and goodness of fit (R^2^) were genuine or occurred by chance (Figure S8, Supplementary Materials). The results of the permutation analysis supported the validity of the original models, as all permuted Q^2^ values were lower than the corresponding original values, and the regression line of the Q^2^ values intersected the vertical axis below zero (Figure S8, Supplementary Materials).

The multivariate findings were complemented by univariate statistical testing (Student’s *t*-test or Mann–Whitney U test, depending on data distribution). The most extensive metabolic dysregulation was observed in the comparison between the MDD and TRD groups (Table 3; Table S2, Supplementary Materials). Consistent with the OPLS-DA models, the TRD group exhibited a more distinct metabolic profile relative to healthy controls than the MDD group did, with the latter showing only a limited number of significantly altered metabolites, primarily within the classes of carboxylic acids and glycerophospholipids (Table 3). Following FDR correction, two metabolites retained robust statistical significance, 2-hydroxyphenylacetic acid and lysophosphatidylethanolamine (LPE) 16:0 (Table 3; Table S2, Supplementary Materials). 2-Hydroxyphenylacetic acid levels were significantly lower in TRD compared to healthy subjects, yet were found to be increased in TRD when compared directly to the MDD group (Table 3). LPE 16:0 exhibited divergent trends. This lipid species was decreased in MDD patients relative to healthy subjects, but showed a significant upward trend in the TRD group (Table 3).

A broader comparison between TRD and healthy controls revealed a significant enrichment of several glycerophospholipids, glycerolipids, and fatty acyls in the TRD group (Table 3). This lipidomic signature was similarly observed when comparing TRD patients to the MDD group. Conversely, organooxygen compounds were consistently less abundant in the TRD cohort compared to both healthy and MDD subjects (Table 3), marking these as potential metabolic hallmarks of treatment resistance.

### 3.3. Treatment-Induced Changes in EV Metabolite Composition in Patients with MDD/TRD

#### 3.3.1. Duloxetine Treatment

To evaluate the biochemical impact of duloxetine, we analyzed longitudinal shifts in the plasma EV metabolome of MDD patients (N = 30) following an 8-week protocol. PCA was performed to assess the unsupervised separation between MDD patients before and after 8 weeks of treatment (Figure S5, Supplementary Materials). Following PCA, supervised OPLS-DA models were constructed to distinguish the two sample groups, and variables with the highest discriminative power were identified through VIP scoring (Table 4). The OPLS-DA models demonstrated a modest separation between subjects at baseline and after duloxetine treatment (Figure S5, Supplementary Materials). Permutation testing (200 iterations) was performed to validate all OPLS-DA models by randomly shuffling class labels, thereby assessing whether the observed predictive ability (Q^2^) and goodness of fit (R^2^) were genuine or occurred by chance (Figure S9, Supplementary Materials).

Our longitudinal analysis revealed that duloxetine therapy significantly modulates the metabolism of carboxylic acids, fatty acyls, and specific lipid species, including two distinct glycerophospholipids (lysophosphatidylcholine (LPC) 18:1 and phosphatidylinositol (PI) 14:1/26:2), the diacylglycerol DAG O-8:0/28:3, 3-hydroxyanthranilic acid, and urate (Table 4). The most pronounced therapeutic shifts were observed in the decreased abundances of leucine, oxalic acid, and DAG O-8:0/28:3 and in the increased abundance of 3-hydroxyanthranilic acid. Notably, the reduction in leucine levels appears to represent a normalization of the metabolic state, as this amino acid was significantly elevated in MDD patients relative to healthy controls at baseline (Table 3). Furthermore, we observed a non-significant increase in the levels of tryptophan and phenylacetylglutamine following treatment (Table 4). Similarly, 3-hydroxyanthranilic acid, which was significantly decreased in both MDD and TRD cohorts at baseline, showed a significant increase following duloxetine administration (Table 4), marking it as a potential biomarker of treatment response. Both LPE 18:1 and urate demonstrated increased abundance post-treatment, though these associations did not survive FDR correction. These specific metabolites were also found to be elevated in the TRD group relative to both healthy controls and MDD patients at baseline (Table 3). Finally, the glycerolipid DAG O-8:0/28:3, which was identified as sensitive to duloxetine treatment, did not show significant baseline alterations in the MDD or TRD groups when compared to healthy controls, suggesting these shifts may be direct pharmacological effects of the medication rather than signatures of disease recovery.

#### 3.3.2. BLT

To investigate the systemic biochemical effects of a non-pharmacological chronotherapeutic intervention, we examined longitudinal changes in the plasma EV metabolome of MDD patients (N = 30) following an 8-week adjunctive BLT protocol. Initially, an unsupervised PCA was performed to assess the global metabolic separation between MDD patients before and after 8 weeks of BLT (Figure S6, Supplementary Materials). Subsequently, supervised OPLS-DA models were constructed to distinguish the two groups, with the most discriminative variables identified via VIP scoring (Table 5). These OPLS-DA models demonstrated a modest but consistent separation between baseline and post-treatment metabolic states (Figure S6, Supplementary Materials). Permutation testing (200 iterations) was performed to validate all OPLS-DA models by randomly shuffling class labels, thereby assessing whether the observed predictive ability (Q^2^) and goodness of fit (R^2^) were genuine or occurred by chance (Figure S9, Supplementary Materials).

Our longitudinal analysis revealed that BLT significantly modulates glycerophospholipid metabolism, while also affecting the abundance of arachidonic acid, 1-monopalmitin, mannose, and cholesterol (Table 5). The most significant alterations were observed in the abundance of 1-monopalmitin and several lysophosphatidylcholines (LPC 16:0, LPC 18:0, LPC 18:3, LPC 20:4, and LPC 20:3). All identified LPCs significantly decreased following BLT (Table 5). This represents a restorative shift, as the abundance of these LPCs was significantly higher in both TRD and MDD subjects compared to healthy controls at baseline (Table 3). Similarly, 1-monopalmitin levels, which were elevated in both MDD cohorts at baseline, showed a significant decrease following the 8-week BLT intervention (Table 5). Furthermore, while the statistical significance for arachidonic acid was attenuated after FDR correction, it is noteworthy that BLT nominally decreased the abundance of this polyunsaturated fatty acid. This is particularly relevant as arachidonic acid levels were significantly elevated in the TRD group compared to both healthy controls and the MDD cohort at baseline (Table 3).

#### 3.3.3. Esketamine Treatment

To evaluate the metabolic shifts associated with a rapid-acting antidepressant intervention, we analyzed longitudinal changes in the plasma EV metabolome of patients with TRD (N = 34) following an 8-week adjunctive intranasal esketamine treatment. Unsupervised PCA was initially performed to assess the global metabolic separation between TRD patients at baseline and following 8 weeks of treatment (Figure S7, Supplementary Materials). Subsequently, supervised OPLS-DA models were constructed to distinguish the two groups, with the most discriminative variables identified via VIP scoring (Table 6). These OPLS-DA models demonstrated a modest but consistent separation between baseline and post-treatment metabolic states (Figure S6, Supplementary Materials). Permutation testing (200 iterations) was performed to validate all OPLS-DA models by randomly shuffling class labels, thereby assessing whether the observed predictive ability (Q^2^) and goodness of fit (R^2^) were genuine or occurred by chance (Figure S9, Supplementary Materials).

Combined results from univariate and multivariate statistical approaches suggest that esketamine treatment significantly modulates fatty acid metabolism (Table 6). Leucic acid levels significantly increased after 8 weeks of treatment, a result that remained robust even after FDR correction (Table 6). Furthermore, the abundance of propionic acid was significantly higher post-treatment compared to baseline, and this finding remained significant after FDR correction (Table 6). This is particularly relevant as propionic acid was found to be significantly less abundant in TRD patients relative to both healthy controls and the MDD group at baseline (Table 3). Esketamine treatment also had a positive effect on the levels of disaccharide (Hex-Hex) (Table 6), which was found to be lower in the TRD group compared to MDD and healthy control subjects at baseline (Table 3). Similarly, TRH, which was significantly decreased in the TRD cohort at baseline, showed a marked increase following esketamine treatment, with the significance surviving FDR correction (Table 6).

A comprehensive overview of the main metabolomic alterations and treatment-induced changes is provided in Figure S10 (Supplementary Materials).

## 4. Discussion

### 4.1. Differential Profiling of EV-Derived Metabolites Across Healthy Controls, MDD, and TRD Patients

The present study provides a comprehensive characterization of the plasma EV metabolome in patients with MDD, TRD, and healthy controls. By applying a multiplatform untargeted metabolomics approach combining GC-MS and LC-MS in both positive and negative ionization modes, a broad range of metabolites were successfully captured. Integration of multivariate and univariate statistical analyses confirmed that the largest degree of metabolic dysregulation occurred between the MDD and TRD groups (Table 3). In contrast, comparisons between MDD and healthy controls revealed fewer significantly altered metabolites, primarily belonging to carboxylic acids and glycerophospholipids (Table 3). This pattern further emphasizes the observations that metabolic disturbances become more pronounced in the context of treatment resistance [23]. Also, the subtle differences between the MDD group and healthy controls may originate from the fact that enrolled patients were partial responders to prior SSRI or SNRI treatment. These findings are summarized schematically in Figure S10 (Supplementary Materials), highlighting the main differences between healthy subjects and patient groups, along with treatment-specific effects.

Across all comparisons, 41 metabolites showed significant changes in relative abundance (Table 3), encompassing several chemical classes such as benzene derivatives, organooxygen compounds, carboxylic acids, fatty acyls, glycerolipids, and glycerophospholipids. As mentioned before, comparison between healthy controls and MDD subjects revealed a shift in amino acid metabolism, in the abundance of several glycerophospholipids, benzene derivatives, and 4-hydroxybenzaldehyde (Table 3). Following FDR correction, two metabolites remained statistically significant, 2-hydroxyphenylacetic acid and LPE 16:0. Altered levels of 2-hydroxyphenylacetic acid may reflect disruptions in aromatic amino acid metabolism, particularly pathways associated with phenylalanine degradation. In case of both the MDD cohort and TRD subjects, a decreased abundance was detected for 2-hydroxyphenylacetic acid (Table 3). When comparing two patient groups, the level of 2-hydroxyphenylacetic acid was lower in the MDD group compared to TRD subjects (Table 3).

Alterations in lipid metabolism have been consistently associated with depression. Liu and colleagues reported elevated levels of several lipid species in depressed individuals, including LPC, LPE, PC, PE, phosphatidylinositol (PI), DG, and TG [24,25]. Previous lipidomic studies have similarly reported elevated plasma LPC species (16:0, 16:1, 18:1, and 22:4) in individuals with depression [26]. In our study, LPE 16:0 and LPC 18:1 were found to be less abundant in the MDD cohort compared to healthy controls, while LPC 18:2 showed a similar trend observed in the TRD group (Table 3). A broader comparison between TRD and healthy individuals revealed an enrichment of several lipid classes, including glycerophospholipids, glycerolipids, and fatty acyls in the TRD group (Table 3). We detected several LPC species that were more abundant in TRD subjects compared to healthy controls and the MDD cohort, while only LPC 18:1 showed a significant decrease (Table 3). We also observed increased levels of several LPE species in the TRD group compared to both healthy controls and the MDD group (Table 3). Altered LPE levels have previously been reported in patients with MDD and, together with LPCs, may represent a lipidomic signature associated with impaired lipid remodeling and reduced cell membrane stability in depression [24,25]. In the TRD group, there was also a significant increase in the abundance of specific glycerolipids, including 1- and 2-monopalmitin and 1- and 2-monostearin (Table 3). These metabolites have been reported to be altered in individuals with depression and in patients treated with SSRIs [27].

We also detected two metabolites related to purine metabolism, hypoxanthine and urate, which were differentially abundant in TRD subjects (Table 3). Previous metabolomic studies have linked depression with alterations in purine metabolism, often characterized by increased hypoxanthine and decreased uric acid levels [10,11], which is opposite to our observations.

Galactose was significantly decreased in TRD subjects compared to healthy controls and MDD patients (Table 3). Consistent with this, Guo et al. reported alterations in metabolites related to purine and galactose metabolism in depressed older adults [28]. As an essential carbohydrate, galactose plays a key role in cellular metabolism and glycosylation and is a component of EV glycoproteins and glycolipids [29,30]. 

Tryptophan, a precursor of serotonin, is metabolized via the kynurenine pathway into compounds such as 3-hydroxyanthranilic acid [31]. In our study, we detected lower levels of both 3-hydroxyanthranilic acid and tryptophane in TRD subjects compared to healthy individuals and MDD group (Table 3). In MDD patients, we detected a trend towards lower levels of tryptophan compared to healthy controls, Similar observations in tryptophane metabolism have been previously associated with depression [32,33].

Fatty acyls, particularly polyunsaturated and saturated fatty acids, have been implicated in depression. We observed increased levels of myristic acid (saturated fatty acid) and two polyunsaturated fatty acids (PUFAs), arachidonic acid and eicosapentaenoic acid, in TRD patients (Table 3). Our results are consistent with findings by da Silva Sabião et al. [34], who reported a higher ratio of polyunsaturated to other fatty acids in depression. Increased levels of different PUFAs were reported in a recent study by Wuang and colleagues [35]. A high ratio of arachidonic acid to eicosapentaenoic acid, an omega-6 fatty acid, was positively correlated with the severity and duration of major depression [35,36]. Our results suggested higher levels of oxononanoic acid in TRD subjects compared to healthy and MDD groups. Elevated levels of oxidative stress and pro-inflammatory mediators, like those triggered by oxononanoic acid, are common biological hallmarks found in individuals with MDD [37]. In our study, the TRD group also had a significantly lower abundance of butyric acid in EVs, compared to the MDD group and healthy individuals (Table 3). Butyric acid is a four-carbon straight short-chain fatty acid found in the human gut [38]. Reduced levels of short-chain fatty acids, specifically butyrate, acetate, and propionate, were linked to depression [39]. 2-Hydroxyisocaproic acid, or leucic acid, is a leucine metabolite and a branched-chain fatty acid. In the TRD group, we detected a higher abundance of leucic acid compared to the healthy control and MDD group. The levels of branched-chain amino acids, including leucine, were reported to be significantly decreased in MDD patients compared to healthy subjects [40].

The results suggest reduced TRH levels in TRD patients (Table 3), which is consistent with its established role in depression and its utility as a neuroendocrine marker for predicting and monitoring treatment response [41].

The abundance of a few metabolites was found to be significantly different only between the two patient groups (MDD vs. TRD). These metabolites include indoline, phosphoric acid, octopamine, and hexanoic acid (Table 3). Octopamine, which was found to decrease in TRD compared to the MDD group, acts as a signaling molecule in the brain that regulates astrocyte metabolism, and deficient production of trace amines, specifically tyramine and octopamine, was reported in subjects diagnosed with depression [42]. Hexanoic acid (caproic acid) is a medium-chain fatty acid often produced by gut microbiota or the diet that was found to correlate with depressive scores [43]. Our results suggest a higher abundance of hexanoic acid in TRD subjects. Indoline is a chemical derivative of indole, a tryptophan metabolite [44]. Its specific role and direct connection to depression remain unconfirmed. Finally, we detected a higher level of phosphate in TRD compared to MDD subjects (Table 3). Harper and colleagues reported alterations in high-energy phosphate metabolism and oxidative phosphorylation regulation in MDD subjects [45], supporting the hypothesis that MDD is associated with the dysfunction of mitochondrial energy metabolism [8].

### 4.2. Treatment-Induced Changes in EV Metabolite Composition in Patients with MDD/TRD

#### 4.2.1. Duloxetine Treatment

Comparison of MDD subjects before and after 8 weeks of duloxetine treatment resulted in a total of ten altered metabolites; however, only four of them remained significant after FDR correction (Table 4). Altered metabolites belonged to the class of carboxylic acids and their derivatives, fatty acyls, glycerolipids, glycerophospholipids, benzene derivatives, and imidazopyrimidines. In the case of amino acids, we detected a decrease in the level of leucine, which was nominally increased in MDD subjects before therapy, compared to healthy controls (Table 3). A deprivation of leucine was found to have a significant protective effect on depression-like behaviors in a mouse model [46]. The results are in line with the idea that antidepressant treatment can lead to a decrease in the levels of branched-chain amino acids, including leucine, and that lower levels of branched-chain amino acids correlate with better treatment outcomes [47]. For tryptophan and phenylacetylglutamine, the treatment effect was opposite to that observed for leucine, and only nominally significant. Similarly, 3-hydroxyanthranilic acid, which was reduced in the MDD and TRD groups at baseline, increased following duloxetine treatment. The glycerophospholipid LPC 18:1 was found to be significantly less abundant in the MDD and TRD cohorts. After duloxetine treatment, its levels increased; however, this change did not reach statistical significance after FDR correction. Another glycerophospholipid that may potentially be influenced by duloxetine therapy is PI 14:1/26:2. The PI content was found to be decreased in a mouse model of depression [48], while antidepressant treatment significantly enhanced phosphoinositide synthesis [49]. In the case of fatty acyls, we detected a significant decrease in the level of oxalic acid (Table 4). The abundance of DAG O-8:0/28:3 decreased after duloxetine treatment. Total levels of DAG lipids were found to be elevated in depression [24,50]. Antidepressant treatment has been shown to decrease the activity of phosphoinositide-specific phospholipase C (PI-PLC), responsible for cleaving membrane phospholipids to produce DAG [51].

#### 4.2.2. BLT

The BLT intervention resulted in significant alterations in the abundance of eleven metabolites, the majority of which belonged to the glycerophospholipid class. All detected glycerophospholipids were lysophospholipids, specifically LPCs and LPEs. The BLT approach exerted a consistent effect across these metabolites, leading to a reduction in the levels of all identified LPCs and LPEs after 8 weeks of treatment. At baseline, several glycerophospholipids, namely LPC 16:0, LPC 20:4, and LPC 20:3, were elevated compared to healthy controls in both the MDD and TRD groups (Table 3). In contrast, the abundance of LPC 18:1 and LPE 16:0 was decreased in MDD patients relative to controls, yet showed higher levels in TRD subjects compared to the MDD group. LPC 18:0 and LPC 18:3 did not differ significantly between baseline patient groups and healthy controls; however, BLT induced a similar decrease in their abundance, consistent with the overall trend observed for glycerophospholipids in plasma EVs (Table 5). In addition to glycerophospholipids, BLT also appeared to affect the levels of arachidonic acid, a polyunsaturated fatty acid that was elevated in both the MDD and TRD groups prior to treatment (Table 3). However, this effect did not reach statistical significance after FDR correction. A similar trend was observed for 1-monopalmitin. BLT could have a negative effect on mannose levels in EVs but this effect did not remain significant after correction for multiple testing. Finally, following BLT, cholesterol levels in EVs increased, although this effect did not remain significant after FDR correction. Lower serum total cholesterol and low-density lipoprotein cholesterol levels have previously been associated with depression [52,53].

#### 4.2.3. Esketamine Treatment

After 8 weeks of treatment with esketamine, a total of seven metabolites were identified as potentially altered by this therapeutic approach in the TRD group. These included three fatty acyls, propionic acid, 1-monopalmitin, and stearic acid, as well as oxalic acid, TRH, and a disaccharide composed of two hexose units (Table 6). However, only four of these compounds remained significant after FDR correction (Table 6). Propionic acid was previously found to be significantly decreased in TRD patients at baseline compared to both healthy controls and MDD patients (Table 3). Following treatment, its levels increased significantly. The potential effect of esketamine on 1-monopalmitin mirrored that observed in MDD patients following BLT, indicating a consistent directional change across treatments. However, it has to be pointed out that in the case of esketamine treatment, this effect did not remain statistically significant after FDR correction. The disaccharide (Hex–Hex) which was decreased in TRD patients at baseline relative to healthy individuals showed a significant increase after treatment. The results also indicate that esketamine may reduce oxalic and stearic acid levels. Leucic acid was elevated in TRD subjects at baseline compared to both healthy controls and MDD patients, and its levels increased further following treatment. Finally, TRH levels, which were significantly reduced in TRD patients at baseline, showed a marked increase after treatment. This finding suggests a restoration of neuroendocrine function, particularly within the hypothalamic–pituitary–thyroid axis, and supports its involvement in the therapeutic effects of esketamine.

### 4.3. Limitations and Strengths of the Study

This study has several notable strengths. The application of a comprehensive multiplatform approach, combining GC-MS and LC-MS, enabled broad metabolite coverage, capturing both polar metabolites and diverse lipid species. The focus on EVs provides a biologically relevant matrix that may better reflect intercellular communication and disease-related processes. Additionally, the inclusion of clinically well-characterized cohorts spanning healthy controls, MDD, and TRD allowed for the identification of severity-specific metabolic alterations. The longitudinal design, incorporating multiple treatment modalities including duloxetine, BLT, and esketamine, further enabled the assessment of dynamic metabolic changes in relation to clinical improvement and the identification of convergent biochemical pathways associated with treatment response.

However, several limitations should be considered. Although the sample size was determined based on an a priori power calculation, inclusion of a larger cohort would further improve statistical power and the generalizability of the findings. In addition, the study population exhibited a relatively low representation of male participants, which may limit the ability to detect sex-specific effects and should be addressed in future studies. Prior exposure to antidepressant medications and the relatively short washout period may have influenced baseline metabolomic profiles, potentially attenuating differences between patient groups and healthy controls. Another limitation that should be pointed out is that the longitudinal analyses were restricted to patients who achieved a clinical response, precluding direct comparison with non-responders. Furthermore, the untargeted nature of the metabolomics approach precludes causal inference and warrants validation in independent cohorts using targeted methods. Finally, the heterogeneous cellular origin of EVs may introduce additional variability and limit precise biological interpretation. Specifically, we cannot confirm whether the EVs are exclusively derived from the central nervous system (CNS), nor can we estimate the relative contribution of CNS- versus periphery-derived EVs. As a result, peripheral sources may have influenced the observed findings and should be considered when interpreting the results.

## 5. Conclusions

This study demonstrates that MDD, particularly its treatment-resistant form, is associated with distinct alterations in EV metabolomic profiles, with the most pronounced dysregulation observed in TRD patients. The applied multiplatform metabolomics approach revealed that lipid metabolism, amino acid pathways, energy homeostasis, and gut–brain axis-related metabolites are significantly affected in depression. Importantly, all therapeutic interventions, duloxetine, BLT, and esketamine, led to partial normalization of metabolic disturbances. Overall, these findings suggest that depression, particularly treatment-resistant depression (TRD), may be associated with systemic metabolic dysregulation and point to extracellular vesicle (EV) metabolomics as a promising platform for the identification of candidate biomarkers of disease and treatment response, pending further validation.

## Figures and Tables

**Table 1:** Demographic and clinical characteristics of healthy control subjects (HC) and patients, divided into two cohorts: patients eligible for duloxetine or BLT (MDD), and patients with treatment-resistant depression (TRD) eligible for treatment with esketamine.

Demographic and Clinical Parameters	HC N = 50	MDD N = 60	TRD N = 65	Test Statistics
Sex (female/male)	40/10	49/11	50/15	Χ^2^ = 3.90; *p* = 0.142
Age (years)	40 (29–69)	51 (31–63)	54 (23–69)	H = 5.71; *p* = 0.058
HAMD-17 score (baseline)	NA	17 (12–27)	21 (19–26)	U = 155.0; *p* = 0.004
MADRS score (baseline)	NA	18 (13–23)	29 (20–32)	U = 331.5; *p* < 0.001

Categorical data was analyzed with the Chi-square test (df = 2). Numerical data were analyzed with the Mann–Whitney U test or Kruskal–Wallis H-test, and shown as median (range). HAMD-17, the Hamilton Depression Rating Scale-17; MARDS, Montgomery–Åsberg Depression Rating Scale; N, number of subjects.

**Table 2:** Demographic and clinical characteristics of MDD patients, divided into three cohorts depending on the therapy approach: patients who were treated with duloxetine (DUL), patients receiving bright-light therapy (BLT), and patients with TRD treated with esketamine (ESK).

Demographic and Clinical Parameters	MDD (DUL) N = 30	MDD (BLT) N = 30	MDD (ESK) N = 34	Test Statistics
Sex (female/male)	23/7	26/4	22/12	Χ^2^ = 3.54; *p* = 0.170
Age (years)	51 (32–62)	51 (31–63)	49 (23–69)	H = 0.47; *p* = 0.792
	HAMD-17 score			
Baseline	17 (12–25)	18.5 (17–27)	22 (20–26)	H = 8.98; *p* = 0.011
Post-TX	8.5 (5–19)	11 (8–13)	9 (4–19)	H = 1.36; *p* = 1.000
Baseline vs. Post-TX	Z = 0.00; *p* < 0.001	Z = 0.00; *p* = 0.028	Z = 0.00; *p* = 0.001	
	MADRS score			
Baseline	18 (13–23)	20 (15–23)	29.5 (20–32) ^a^	H = 19.62; *p* < 0.001
Post-TX	10.5 (5–19)	12 (6–17)	11 (4–26)	H = 1.87; *p* = 0.939
Baseline vs. Post-TX	Z = 0.00; *p* < 0.001	Z = 0.00; *p* = 0.042	Z = 0.00; *p* = 0.001	

Categorical data was analyzed with the χ^2^ test (df = 2). Numerical data were analyzed with the Kruskal–Wallis ANOVA on ranks, or, in case of paired samples, the Wilcoxon signed-rank test, and shown as median (range). HAMD-17, the Hamilton Depression Rating Scale-17; MARDS, Montgomery–Åsberg Depression Rating Scale; N, number of subjects; TX, therapy. ^a^ *p* < 0.05; Dunn’s post hoc test, MDD (ESK) vs. MDD (DUL) and MDD (ESK) vs. MDD (BLT).

**Table 3:** Differential metabolites detected by GC-MS and LC-MS analysis in plasma EVs of healthy controls (HC), patients with major depressive disorder (MDD) or with diagnosed treatment-resistant depression (TRD).

Class	Compound	Platform (Mode)	HC vs. MDD			HC vs. TRD			MDD vs. TRD		
			*p*	VIP	Log_2_FC	*p*	VIP	Log_2_FC	*p*	VIP	Log_2_FC
Benzene derivatives	2-Hydroxyphenylacetic acid	LC-MS(−)	**0.005**	1.06	−2.88	**0.004**	1.35	−1.56	**0.001**	1.00	1.32
	3-Hydroxyanthranilic acid	LC-MS(−)	0.023	1.01	−1.11	**<** **0.001**	1.19	−2.30	**<** **0.001**	1.19	−1.19
Carboxylic acids	Propionic acid	LC-MS(+)	NS		0.10	**0.004**	1.47	−0.32	**0.004**	1.21	−0.33
	Leucine	GC-MS	0.031	1.33	0.13	NS		−0.21	NS		−0.33
	Sarcosine	GC-MS	0.040	1.16	0.37	NS		0.25	NS		−0.12
	Phenylalanine	LC-MS(+)	0.036	1.32	−0.55	NS		0.34	0.017	1.27	0.89
	Tryptophan	LC-MS(+)	NS		−0.39	NS		0.40	**0.002**	1.47	0.79
		LC-MS(−)	NS		−2.29	**0.001**	1.18	−1.40	**0.001**	1.47	0.89
	Tryptophan betaine	LC-MS(+)	0.037	1.12	−1.43	NS		−0.52	NS		0.92
	Isoleucylglutamate	LC-MS(+)	NS		−0.41	0.039	1.52	2.56	**0.005**	2.05	2.97
	Phenylacetylglutamine	LC-MS(−)	NS		−1.66	**0.012**	1.00	−1.03	**0.019**	1.01	0.63
Fatty acyls	Hexanoic acid	GC-MS	NS		−0.15	NS		0.32	**0.002**	1.17	0.48
	Myristic acid	GC-MS	NS		−0.04	**<** **0.001**	1.49	0.98	**<** **0.001**	1.31	0.72
	Arachidonic acid	GC-MS	NS		0.24	**<** **0.001**	1.62	1.30	**<** **0.001**	1.48	1.06
	Eicosapentaenoic acid	GC-MS	NS		0.28	**<** **0.001**	1.25	1.28	**<** **0.001**	1.28	1.00
	Butyric acid	LC-MS(+)	NS		−0.09	0.017	1.22	−0.51	**0.004**	1.03	−0.41
	Oxononanoic acid	LC-MS(−)	NS		0.13	**0.006**	1.21	0.22	**0.026**	1.06	0.09
	Leucic acid	LC-MS(−)	NS		−0.13	**0.003**	1.26	0.28	**0.029**	1.01	0.41
Glycerolipids	2-Monopalmitin	GC-MS	NS		0.29	**0.001**	1.23	0.82	**0.002**	1.08	0.53
	1-Monopalmitin	GC-MS	NS		0.31	**<** **0.001**	1.39	1.32	**<** **0.001**	1.24	1.02
	2-Monostearin	GC-MS	NS		0.18	**<** **0.001**	1.12	0.90	**<** **0.001**	1.16	0.75
	1-Monostearin	GC-MS	NS		0.12	**<** **0.001**	1.59	1.44	**<** **0.001**	1.62	1.32
Glycerophospholipids	LPC 16:1	LC-MS(+)	NS		0.25	0.027	1.13	0.59	NS		0.34
	LPC 20:3	LC-MS (+)	NS		0.35	**0.003**	1.12	0.69	**0.003**	1.21	0.35
	LPC 16:0	LC-MS(−)	NS		0.47	**0.008**	1.24	1.06	**0.013**	1.09	0.59
	LPC 18:1	LC-MS(+)	NS		−0.11	NS		0.36	0.037	1.00	0.34
		LC-MS(−)	0.046	1.10	−0.91	0.034	1.22	−0.20	**0.003**	1.18	0.71
	LPC 18:2	LC-MS(+)	NS		−0.29	NS		0.14	0.039	1.03	0.43
		LC-MS(−)	0.046	1.18	0.29	0.041	1.21	0.99	**0.004**	1.25	0.71
	LPC 20:4	LC-MS(−)	NS		0.36	0.032	1.15	0.86	**0.003**	1.19	0.50
	LPE 16:0	LC-MS(−)	**0.012**	1.44	−0.30	**<** **0.001**	1.54	0.83	**0.001**	1.33	1.13
	LPE 18:1	LC-MS(−)	NS		−0.52	**0.005**	1.20	0.48	**0.001**	1.19	1.00
	LPE 18:2	LC-MS(−)	NS		−0.85	**0.004**	1.35	−0.16	**0.001**	1.23	0.69
	LPE 20:4	LC-MS(−)	NS		0.52	**0.001**	1.30	1.48	**0.003**	1.19	0.95
Organooxygen compd.	Galactose	LC-MS(+)	NS		0.05	**0.005**	1.41	−0.29	**0.011**	1.21	−0.34
	Ribulose-5-phosphate	GC-MS	NS		−0.04	**0.001**	1.00	−0.21	NS		−0.17
	Disaccharide (Hex-Hex)	LC-MS(+)	NS		0.07	**0.005**	1.46	−0.30	**0.003**	1.28	−0.37
Imidazopyrimidines	Hypoxanthine	LC-MS(−)	NS		−1.31	**0.003**	1.08	−0.51	**0.003**	1.08	0.80
	Urate	LC-MS(−)	NS		0.59	**0.012**	1.03	1.09	**0.023**	1.08	0.51
Indolines	Indoline	LC-MS(+)	NS		−0.65	NS		0.26	**0.007**	1.27	0.91
Non-metal oxoanions	Phosphoric acid	GC-MS	NS		−0.13	NS		0.16	**<** **0.001**	1.15	0.29
Steroids	TRH	LC-MS(−)	NS		−2.66	**0.001**	1.72	−3.33	**<** **0.001**	1.17	−0.67
Phenols	Octopamine	GC-MS	NS		0.18	NS		−0.38	**<** **0.001**	1.09	−0.60
Hydroxy acids	6-Hydroxycaproic acid	LC-MS(+)	NS		−0.12	NS		−0.50	0.026	1.68	−0.37

Bolded *p*-values indicate results that remained statistically significant after false discovery rate (FDR) correction. GC-MS, gas chromatography coupled to mass spectrometry; Hex, hexose; LC-MS, liquid chromatography coupled to electrospray ionization mass spectrometry; Log_2_FC, log_2_ fold change; LPC, lysophosphatidylcholine; LPE, lysophosphatidylethanolamine; NS, non-significant; *p*, probability value; TRH, thyrotropin-releasing hormone; VIP, variable importance in projection.

**Table 4:** Differential metabolites detected by GC-MS and LC-MS analysis in plasma EVs following 8 weeks of duloxetine (DUL) treatment in MDD patients.

Class	Compound	Platform (Mode)	MZ	RT	Baseline (DUL) vs. Post-TX (DUL)				
					*p*	q	VIP	FC	Log_2_FC
Carboxylic acids	Leucine	GC-MS	86.1	8.04	0.002	0.039	1.75	0.65	−0.62
	Tryptophan	LC-MS(−)	203.0830	4.48	0.039	NS	1.21	1.42	0.50
	Phenylacetylglutamine	LC-MS(−)	263.1038	5.04	0.011	NS	11.58	2.10	1.07
Fatty acyls	Oxalic acid	GC-MS	73.1	8.04	0.002	0.039	1.15	0.66	−0.59
	Azelaic acid	LC-MS(−)	187.0975	6.07	0.017	NS	1.13	0.83	−0.27
Glycerolipids	DAG O-8:0/28:3	LC-MS(+)	622.5774	6.80	0.009	0.034	1.16	0.85	−0.23
Glycerophospholipids	LPC 18:1	LC-MS(−)	566.3465	9.83	0.035	NS	1.23	1.58	0.66
	PI 14:1/26:2	LC-MS(−)	915.5966	13.55	0.037	NS	2.16	1.28	0.35
Benzene deriv.	3-Hydroxyanthranilic acid	LC-MS(−)	152.0356	7.24	0.004	0.037	1.83	1.93	0.95
Imidazopyrimidines	Urate	LC-MS(−)	167.0211	0.94	0.048	NS	1.15	1.26	0.33

DAG, diacylglyceride; FC, fold change; GC-MS, gas chromatography coupled to mass spectrometry; LC-MS, liquid chromatography coupled to electrospray ionization mass spectrometry; Log_2_FC, log_2_ fold change; LPC, lysophosphatidylcholine; MZ, mass-to-charge ratio; NS, non-significant; *p*, probability value; PI, phosphatidylinositol; q, FDR adjusted *p*-value; RT, retention time; TX, therapy; VIP, variable importance in projection.

**Table 5:** Differential metabolites detected by GC-MS and LC-MS analysis in plasma EVs following 8 weeks of bright-light therapy (BLT) in MDD patients.

Class	Compound	Platform (Mode)	MZ	RT	Baseline (BLT) vs. Post-TX (BLT)				
					*p*	q	VIP	FC	Log_2_FC
Fatty acyls	Arachidonic acid	GC-MS	79.0	23.48	0.012	NS	1.22	0.74	−0.44
Glycerolipids	1-Monopalmitin	GC-MS	371.3	23.48	0.001	0.048	1.30	0.65	−0.63
Glycerophospholipids	LPC 16:0	LC-MS(+)	496.3402	9.57	0.016	NS	1.63	0.72	−0.48
		LC-MS(−)	540.3312	9.40	0.010	0.026	1.85	0.77	−0.38
	LPC 18:0	LC-MS(+)	524.3715	10.78	0.003	0.257	1.40	0.63	−0.66
	LPC 18:1	LC-MS(+)	522.3561	9.84	0.024	NS	1.63	0.75	−0.42
		LC-MS(−)	566.3465	9.83	0.029	NS	1.76	0.81	−0.30
	LPC 18:3	LC-MS(+)	518.3225	9.57	0.006	0.026	1.58	0.73	−0.45
	LPC 20:4	LC-MS(−)	588.3310	9.22	0.008	0.026	1.47	0.85	−0.24
	LPE 16:0	LC-MS(−)	452.2785	9.42	0.046	NS	1.77	0.73	−0.45
	LPC 20:3	LC-MS(+)	546.3567	9.43	0.008	0.026	1.45	0.72	−0.48
Organooxygen compd.	Mannose	GC-MS	319.1	17.39	0.039	NS	1.37	0.77	−0.37
Steroids and derivatives	Cholesterol	GC-MS	129.1	27.59	0.048	NS	1.26	1.39	0.48

FC, fold change; GC-MS, gas chromatography coupled to mass spectrometry; LC-MS, liquid chromatography coupled to electrospray ionization mass spectrometry; Log_2_FC, log_2_ fold change; LPC, lysophosphatidylcholine; LPE, lysophosphatidylethanolamine; MZ, mass-to-charge ratio; NS, non-significant; *p*, probability value; q, FDR adjusted *p*-value; RT, retention time; TX, therapy; VIP, variable importance in projection.

**Table 6:** Differential metabolites detected by GC-MS and LC-MS analysis in plasma EVs following 8 weeks of esketamine (ESK) treatment in MDD patients.

Class	Compound	Platform (Mode)	MZ	RT	Baseline (ESK) vs. Post-TX (ESK)				
					*p*	q	VIP	FC	Log_2_FC
Carboxylic acids	Propionic acid	LC-MS(+)	97.0284	0.75	0.009	0.027	1.27	1.19	0.25
Fatty acyls	Oxalic acid	GC-MS	73.1	8.04	0.032	NS	1.48	0.79	−0.34
	Stearic acid	GC-MS	117.0	20.72	0.041	NS	1.19	0.83	−0.27
	Leucic acid	LC-MS(−)	131.0715	5.18	0.002	0.009	2.88	1.68	0.75
Glycerolipids	1-Monopalmitin	GC-MS	371.3	23.48	0.034	NS	1.81	0.69	−0.53
Organooxygen compd.	Disaccharide (Hex-Hex)	LC-MS(+)	325.1127	0.75	0.010	0.027	1.20	1.19	0.26
Steroids	TRH	LC-MS(−)	361.1635	8.31	0.005	0.013	2.13	1.42	0.50

FC, fold change; GC-MS, gas chromatography coupled to mass spectrometry; Hex, hexose; LC-MS, liquid chromatography coupled to electrospray ionization mass spectrometry; Log_2_FC, log_2_ fold change; MZ, mass-to-charge-ratio; NS, non-significant; *p*, probability value; q, FDR adjusted *p*-value; RT, retention time; TRH, thyrotropin-releasing hormone; TX, therapy; VIP, variable importance in projection.

## Data Availability

The data presented in this study are available on request from the corresponding author due to ethical reasons.

## Supplementary Materials

The following supporting information can be downloaded at: https://www.mdpi.com/article/10.3390/biom16040533/s1, Figure S1: Study population and design; Figure S2: Validation and confirmation of isolated plasma EVs; Figure S3: Principal Component Analysis (PCA): differences in EV metabolite profiles between healthy control subjects and MDD patients, divided into two cohorts based on subsequent treatment: the group treated with duloxetine and/or BLT, MDD baseline, and the TRD cohort treated with esketamine; Figure S4: Differences in EV metabolite profiles between healthy control subjects and MDD patients, divided into two cohorts, MDD baseline and TRD baseline; Figure S5: Differences in EV metabolite profiles between MDD patients at baseline and after 8 weeks (Post-TX) of duloxetine (DUL) therapy; Figure S6: Differences in EV metabolite profiles between MDD patients at baseline and after 8 weeks (Post-TX) of bright-light therapy (BLT); Figure S7: Differences in EV metabolite profiles between MDD patients at baseline and after 8 weeks (Post-TX) of esketamine (ESK) therapy; Figure S8: Permutation analysis plotting R2 and Q2 from 200 permutation tests in the OPLS-DA models obtained by comparing EV metabolite profiles between healthy control subjects and MDD patients, divided into two cohorts, MDD baseline and TRD baseline; Figure S9: Permutation analysis plotting R2 and Q2 from 200 permutation tests in the OPLS-DA models obtained by comparing EV metabolite profiles of MDD and TRD patients at baseline and after 8 weeks of adequate therapy (duloxetine, bright-light therapy, or esketamine treatment); Figure S10: Graphical summary of key metabolomic findings and treatment effects; Table S1: Significantly altered compounds, detected by GC-MS and LC-MS analysis in plasma EVs, between healthy controls, patients with major depressive disorder (MDD), or with diagnosed treatment-resistant depression (TRD); Table S2: Differential metabolites detected by GC-MS and LC-MS analysis in plasma EVs of healthy controls (HC), patients with major depressive disorder (MDD) or with diagnosed treatment-resistant depression (TRD).

## Abbreviations

The following abbreviations are used in this manuscript:

MDD	Major depressive disorder
SSRI	Serotonin reuptake inhibitors
SNRI	Serotonin–norepinephrine reuptake inhibitors
TRD	Treatment-resistant depression
BLT	Bright-light therapy
EV	Extracellular vesicles
GC-MS	Gas chromatography coupled with mass spectrometry
LC-MS	Liquid chromatography coupled with mass spectrometry
HAMD-17	Hamilton Depression Rating Scale
MADRS	Montgomery–Åsberg Depression Rating Scale
PBS	Phosphate-buffered saline
NTA	Nanoparticle tracking analysis
TEM	Transmission electron microscopy
ACN	Acetonitrile
PCA	Principal component analysis
OPLS-DA	Orthogonal partial least squares discriminant analysis
VIP	Variable importance in projection
ANOVA	Analysis of variance
FDR	False discovery rate
FC	Fold change
Log2FC	log2 fold change
HC	Healthy controls
N	Number of subjects
LPC	Lysophosphatidylcholine
LPE	Lysophosphatidylethanolamine
PC	Phosphatidylcholine
MZ	Mass-to-charge ratio
Hex	Hexose
p	Probability value
q	FDR adjusted p-value
RT	Retention time
TRH	Thyrotropin-releasing hormone
DAG	Diacylglycerol
PI	Phosphatidylinositol
TG	Triglycerides
