# Supplementary material for: Metabolomic Profiling of Extracellular Vesicles Reveals Distinct Metabolic Dysregulation and Treatment-Specific Signatures in Depression
Source: Biomolecules. 2026 Apr 2;16(4):533. doi: 10.3390/biom16040533 (PMC13113742; doi:10.3390/biom16040533)
Supplement: Supplementary file 1 [file biomolecules-16-00533-s001.zip › biomolecules-4237440-supplementary pub2.pdf]

biomolecules

MDPI

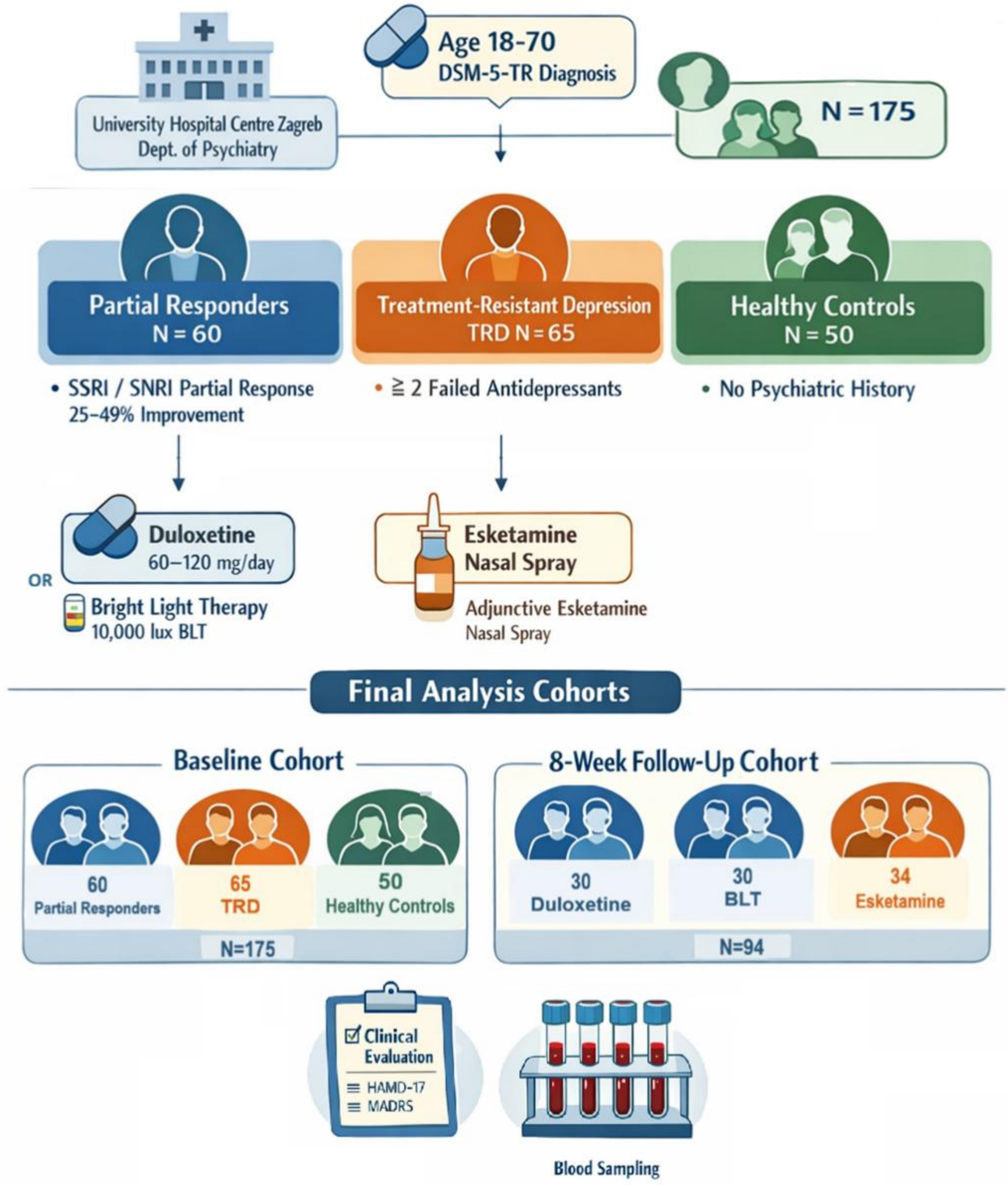

Figure S1. Study population and design. Image generated using ChatGPT (2026, March 15 version), https://chat.openai.com/. DSM-5-TR, Diagnostic and Statistical Manual of Mental Disorders, Fifth Edition, Text Revision; HAMD-17, Hamilton Depression Rating Scale; MADRS, Montgomery–Åsberg Depression Rating Scale; N, Number of participants; TRD, Treatment-resistant depression

 https://doi.org/10.3390/biom16040533

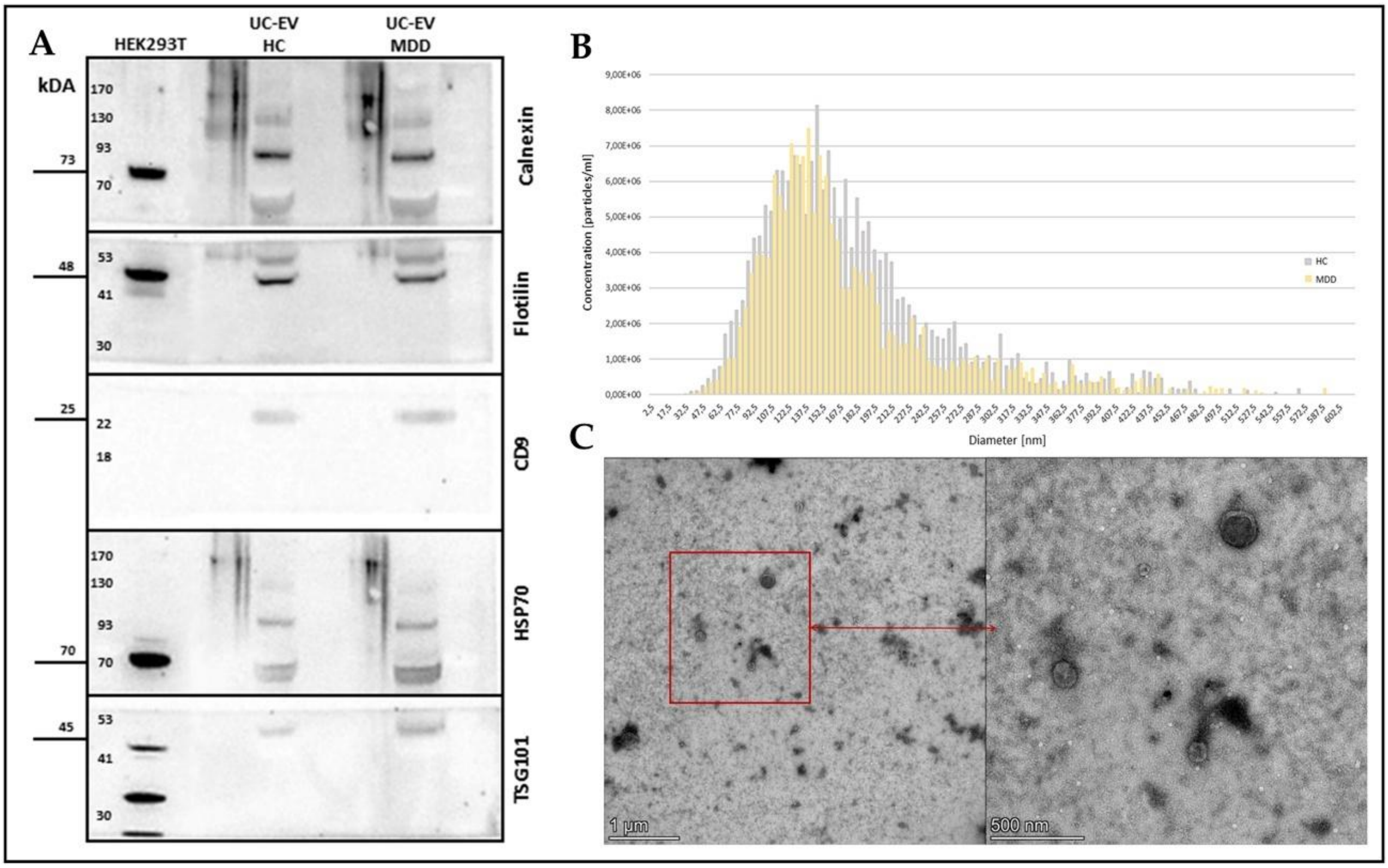

Figure S2. Validation and confirmation of isolated plasma EVs. **(A)** Western blot analysis of plasma EVs and cell lysate from HEK293T as a control. Antibodies against proteins enriched in small EVs (HSP70, flotillin-1, CD9, TSG101), and against proteins of the endoplasmic reticulum (calnexin) were used; (**B)** Graphical presentation of the size of isolated EVs from NTA; (**C)** TEM micrographs of representative plasma EVs. EV, extracellular vesicles; HC, healthy controls; MDD, major depressive disorder; UC, ultracentrifugation.

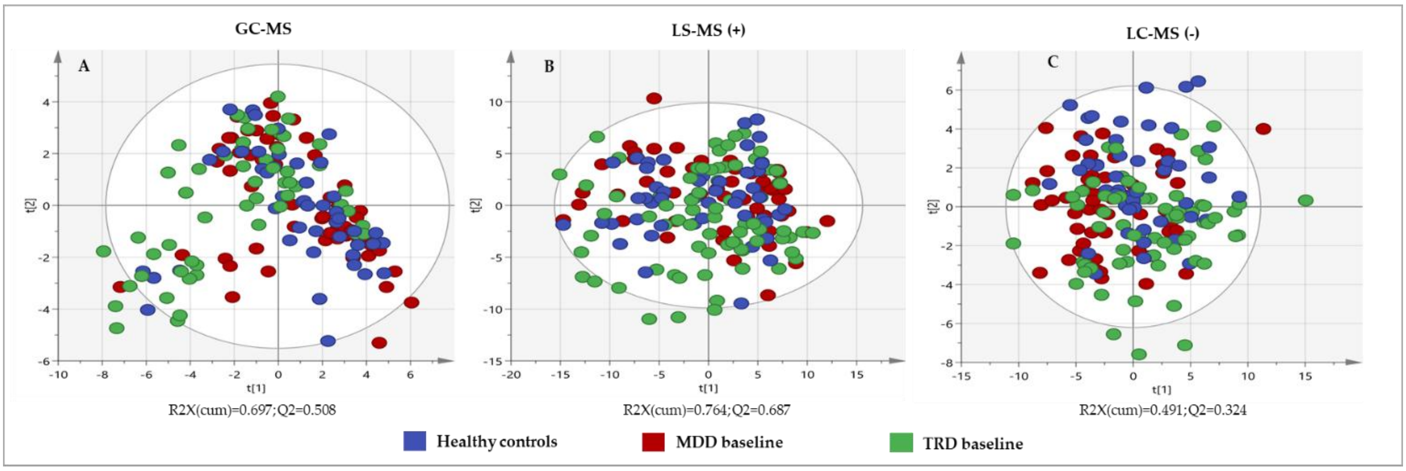

Figure S3. Principal Component Analyses (PCA): differences in EV metabolite profiles between healthy control subjects and MDD patients, divided into two cohorts based on subsequent treatment: the group treated with duloxetine and/or BLT, MDD baseline, and the TRD cohort that will be treated with esketamine. PCA plots were obtained using SIMCA-P+ software (version 15.0.2.5959, Umetrics, Umea, Sweden). **(A)** PCA score plot for the GC-MS analysis; **(B)** PCA score plot for the LC-MS ESI (+) analysis; **(C)** PCA score plot for the LC-MS ESI (-) analysis. Before analysis, the data was log transformed and scaled to Unit Variance (UV scaling).

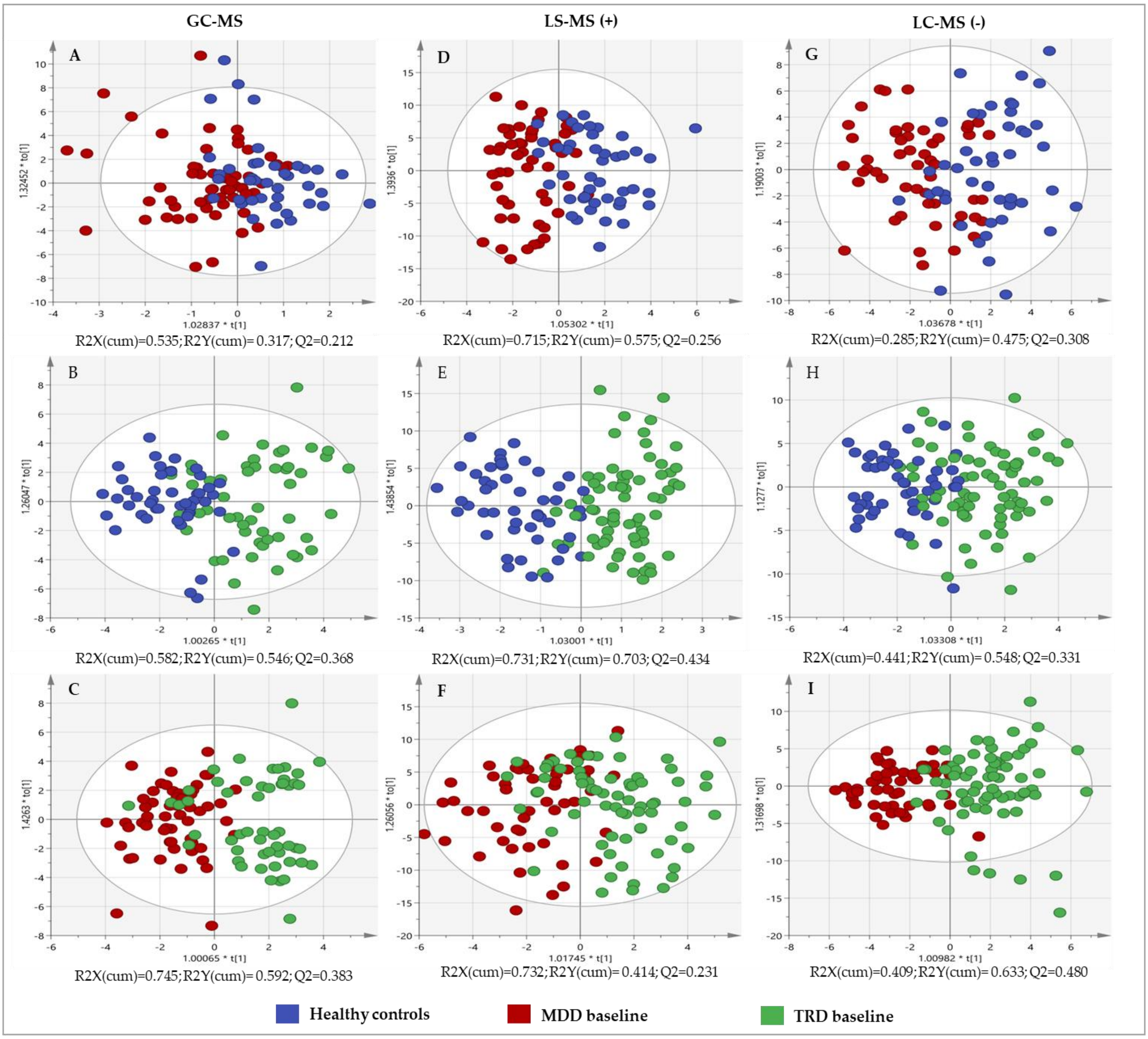

Figure S4. Differences in EV metabolite profiles between healthy control subjects and MDD patients, divided into two cohorts, MDD baseline and TRD baseline. OPLS-DA plots were obtained using SIMCA-P+ software (version 15.0.2.5959, Umetrics, Umea, Sweden). **(A–C)** OPLS-DA score plots for the GC-MS analysis; **(D–F)** OPLS-DA score plots for the LC-MS ESI (+); **(G–I)** OPLS-DA score plot for the LC-MS ESI (-) analysis.

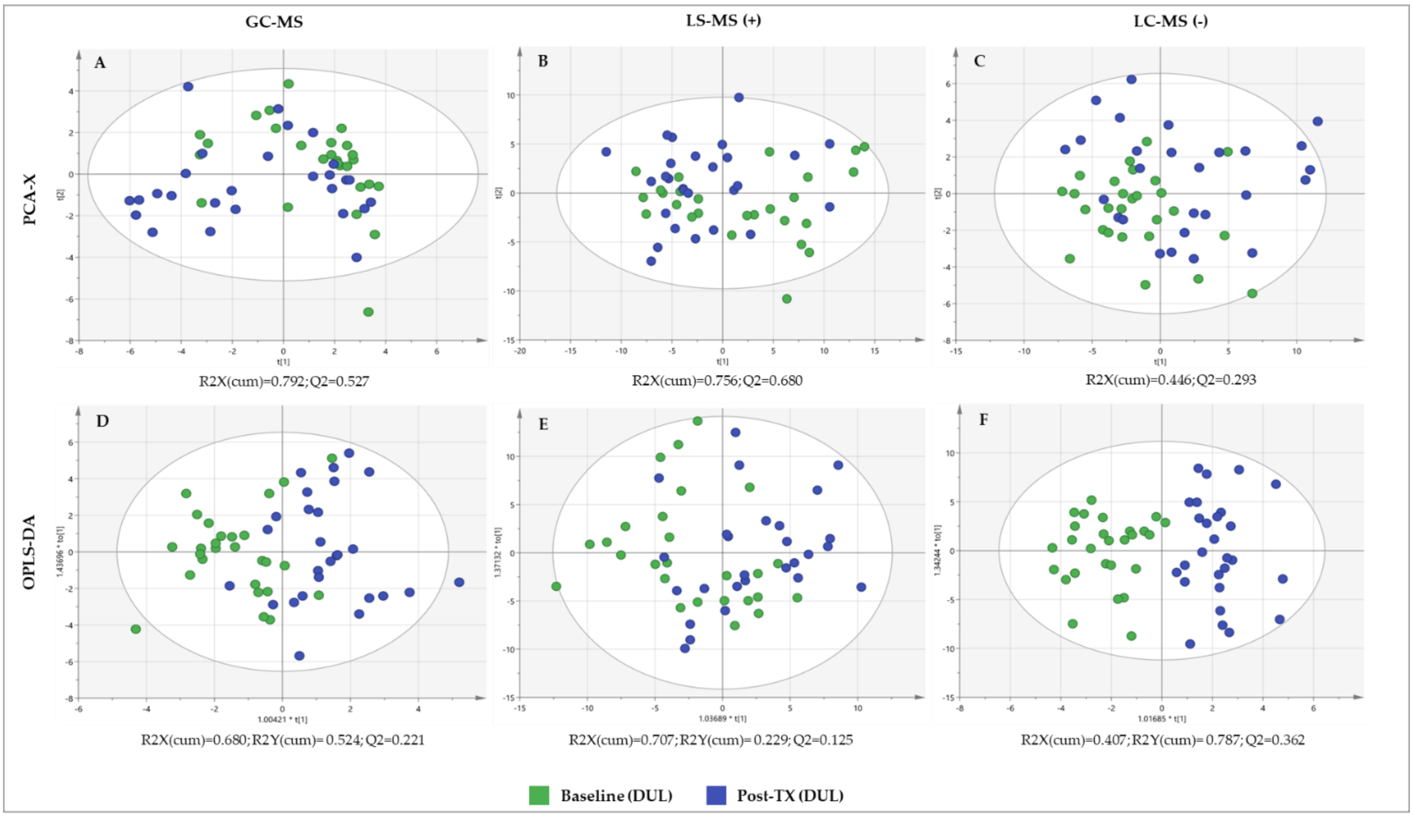

Figure S5. Differences in EV metabolite profiles between MDD patients at baseline and after 8 weeks (Post-TX) of duloxetine (DUL) therapy. PCA and OPLS-DA plots were obtained using SIMCA-P+ software (version 15.0.2.5959, Umetrics, Umea, Sweden). **(A–C)** PCA score plots for the GC-MS, LC-MS ESI (+) mode, and LC-MS ESI (-) mode; **(D–F)** OPLS-DA score plots for the GC-MS, LC-MS ESI (+) mode, and LC-MS ESI (-) mode.

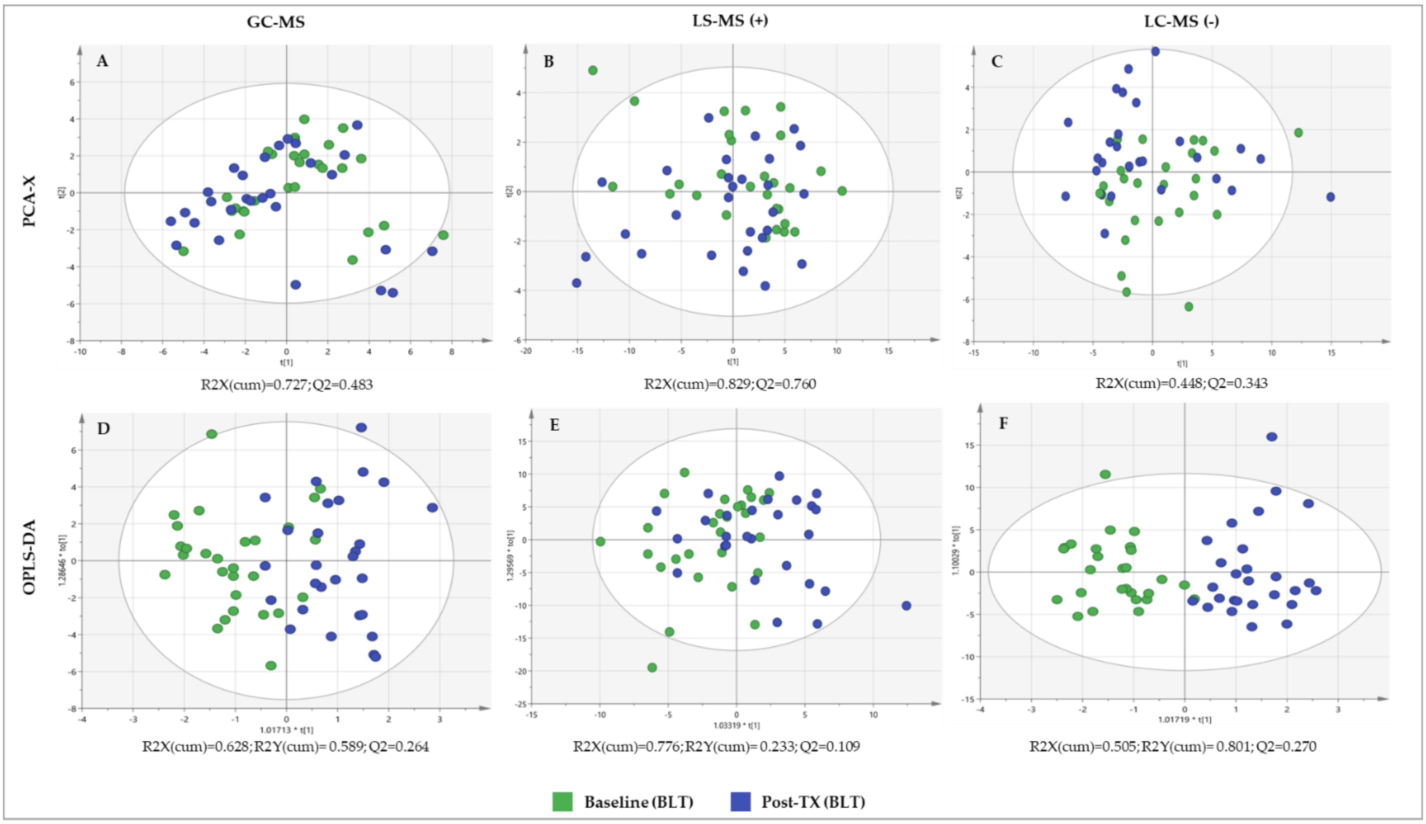

Figure S6. Differences in EV metabolite profiles between MDD patients at baseline and after 8 weeks (Post-TX) of bright-light therapy (BLT). PCA and OPLS-DA plots were obtained using SIMCA-P+ software (version 15.0.2.5959, Umetrics, Umea, Sweden). **(A–C)** PCA score plots for the GC-MS, LC-MS ESI (+) mode, and LC-MS ESI (-) mode; **(D–F)** OPLS-DA score plots for the GC-MS, LC-MS ESI (+) mode, and LC-MS ESI (-) mode.

https://doi.org/10.3390/biom16040533

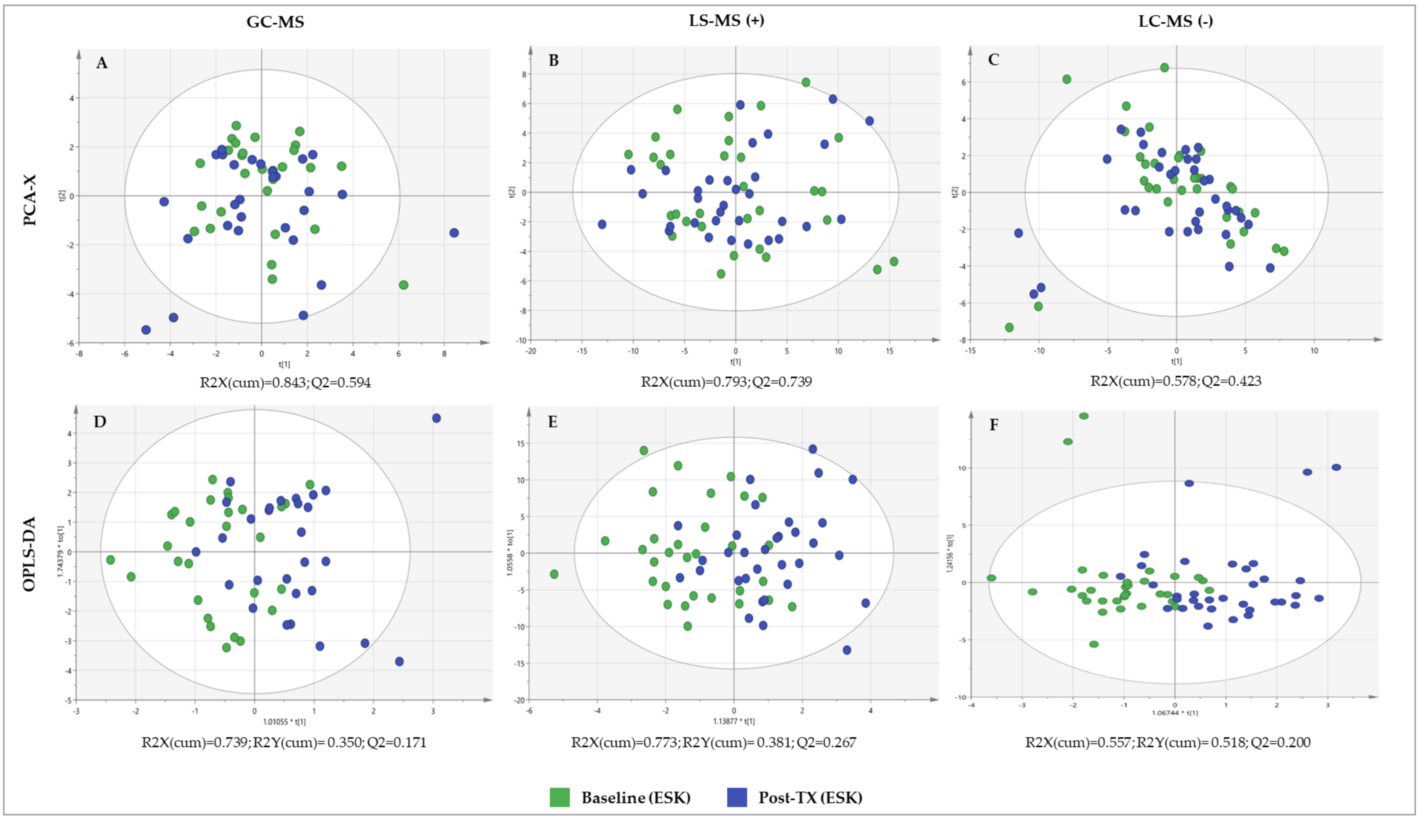

Figure S7. Differences in EV metabolite profiles between MDD patients at baseline and after 8 weeks (Post-TX) of esketamine (ESK) therapy. PCA and OPLS-DA plots were obtained using SIMCA-P+ software (version 15.0.2.5959, Umetrics, Umea, Sweden). **(A) - (C)** PCA score plots for the GC-MS, LC-MS ESI (+) mode, and LC-MS ESI (-) mode; **(D) – (F)** OPLS-DA score plots for the GC-MS, LC-MS ESI (+) mode, and LC-MS ESI (-) mode.

https://doi.org/10.3390/biom16040533

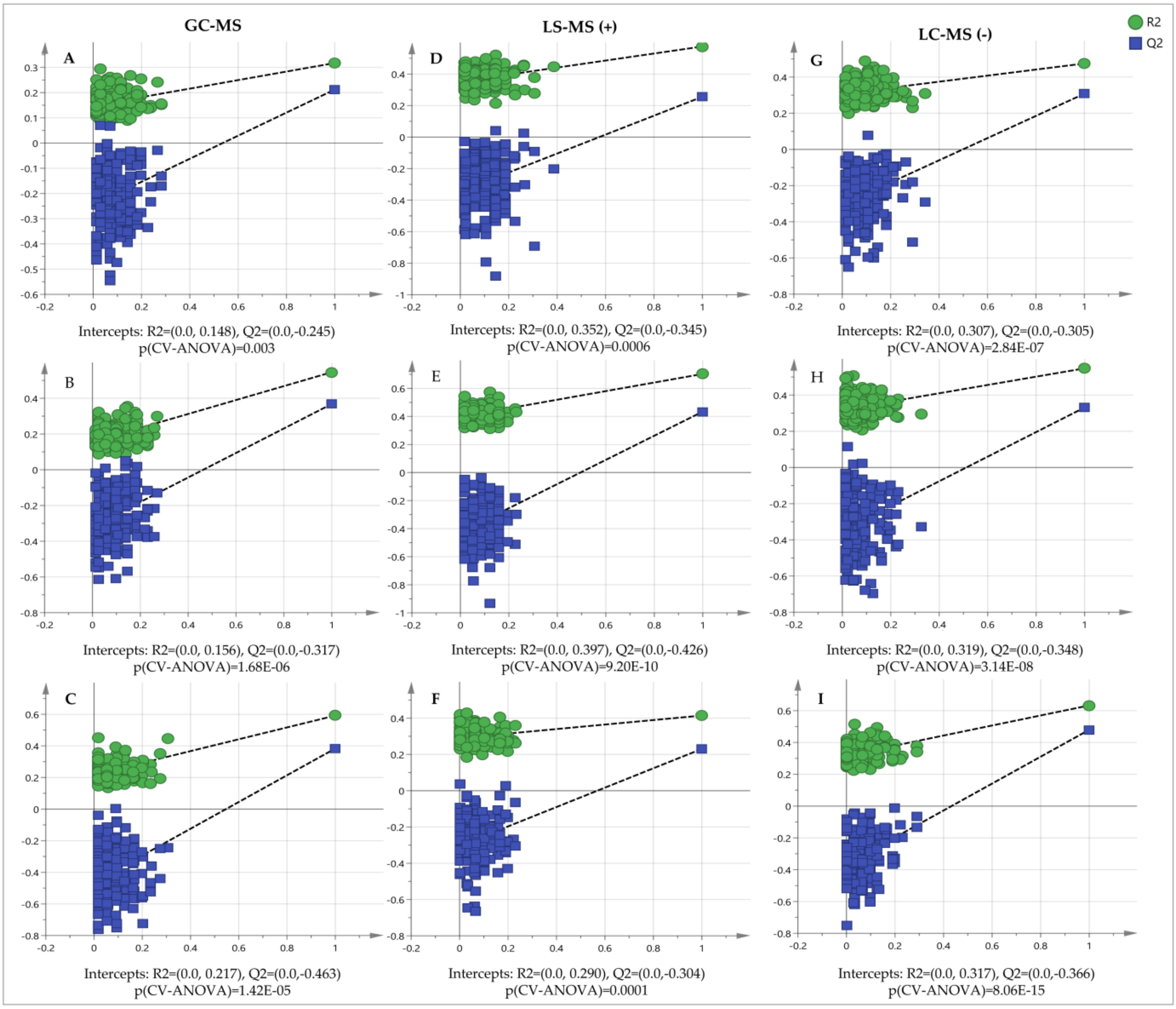

Figure S8. Permutation analysis plotting R2 and Q2 from 200 permutation tests in the OPLS-DA models obtained by comparing EV metabolite profiles between healthy control subjects and MDD patients, divided into two cohorts, MDD baseline and TRD baseline. Plots were obtained using SIMCA-P+ software (version 15.0.2.5959, Umetrics, Umea, Sweden). **(A), (D), (G)** Permutation analyses for OPLS-DA models comparing healthy controls and MDD subjects; **(B), (E), (H)** Permutation analyses for OPLS-DA models comparing healthy controls and TRD subjects; **(C), (F), (I)** Permutation analyses for OPLS-DA models MMD and TRD patients.

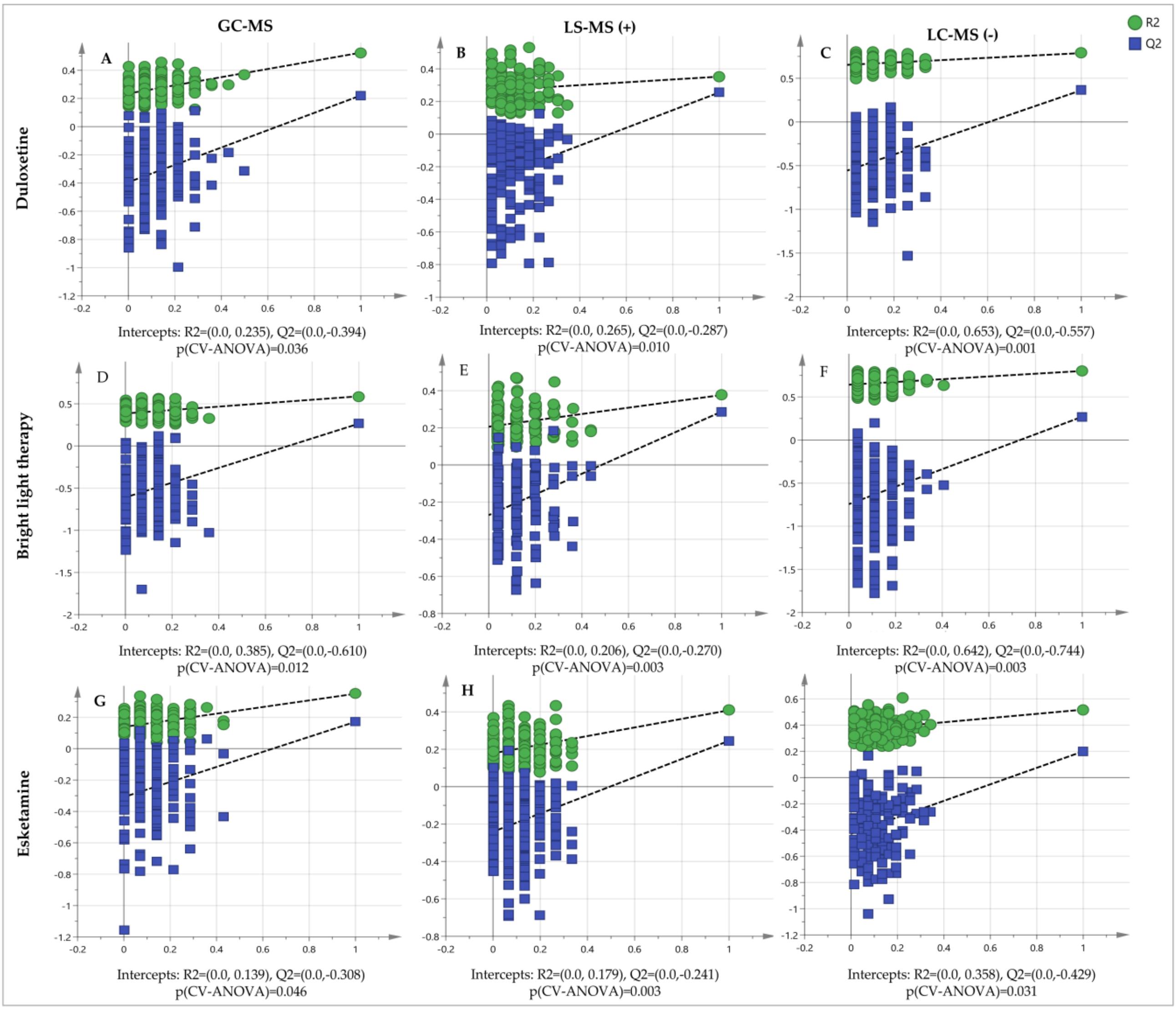

Figure S9. Permutation analysis plotting R2 and Q2 from 200 permutation tests in the OPLS-DA models obtained by comparing EV metabolite profiles of MDD and TRD patients at baseline and after 8 weeks of adequate therapy (duloxetine, bright light therapy, or esketamine treatment)**.** Plots were obtained using SIMCA-P+ software (version 15.0.2.5959, Umetrics, Umea, Sweden). **(A–C)** Permutation analyses for OPLS-DA models comparing MDD subjects before and after duloxetine treatment; **(D–F)** Permutation analyses for OPLS-DA models comparing MDD subjects before and after bright light therapy; **(G–I)** Permutation analyses for OPLS-DA models comparing TRD subjects before and after esketamine treatment.

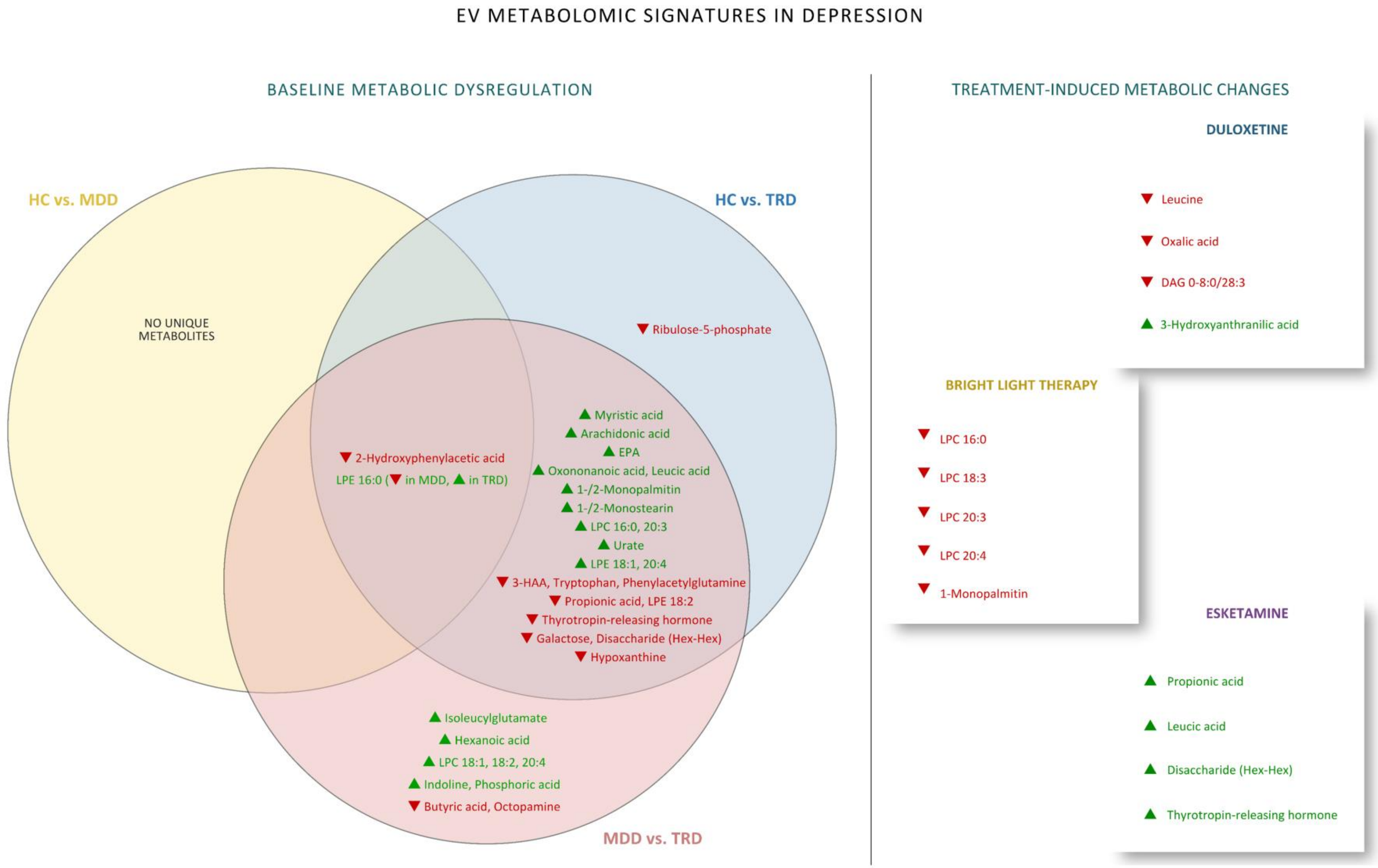

Figure S10. Graphical summary of key metabolomic findings and treatment effects. Metabolites with increased abundance are indicated in green, whereas those with decreased abundance are shown in red (based on the results presented in Tables 3–6). DAG, diacylglyceride; EPA, eicosapentaenoic acid ; HC, healthy controls; 3-HAA, 3-hydroxyanthranilic acid; LPC, lysophosphatidylcholine; LPE, lysophosphatidylethanolamine; MDD, major depressive disorder; TRD, treatment-resistant depression.

Table S1. Significantly altered compounds, detected by GC-MS and LC-MS analysis in plasma EVs, between healthy controls, patients with major depressive disorder (MDD), or with diagnosed treatment-resistant depression (TRD).

Class	Compound	Platform (mode)	RT	MZ	ANOVA/K-W		
					p	q	Significance
Benzene and derivatives	3-Hydroxyanthranilic acid	LC-MS (-)	7.24	152.0356	<0.001	<0.001	b, c
Organooxygen compd.	Disaccharide (Hex-Hex)	LC-MS (+)	0.75	325.1127	0.002	0.037	b, c
	Galactose	LC-MS (+)	0.75	198.0968	0.007	0.049	b, c
	Ribulose-5-phosphate	GC-MS	19.69	115.1	0.001	0.006	b, c
Carboxylic acids and derivatives	Propionic acid	LC-MS (+)	0.75	97.0284	0.004	0.037	b, c
	Tryptophan	LC-MS (-)	4.48	203.0830	0.002	0.006	c
		LC-MS (+)	4.48	205.0971	0.007	0.047	c
	Isoleucylglutamate	LC-MS (+)	4.57	261.1450	<0.001	0.016	b, c
	Phenylacetylglutamine	LC-MS (-)	5.04	263.1038	0.018	0.045	c
Fatty Acyls	Hexanoic acid	GC-MS	7.12	75.0	0.002	0.005	c
	Oxononanoic acid	LC-MS (-)	7.55	173.1186	0.008	0.024	b
	Myristic acid	GC-MS	21.97	343.3	<0.001	<0.001	b, c
	Arachidonic acid	GC-MS	23.48	79.0	0.001	0.007	b, c
	Eicosapentaenoic acid	GC-MS	23.89	361.2	0.005	0.017	b
Glycerolipids	TG O-8:0/16:4/18:5	LC-MS (+)	11.84	713.5136	<0.001	0.009	a, c
	2-Monopalmitin	GC-MS	23.22	129.0	0.004	0.016	c
	1-Monopalmitin	GC-MS	23.48	371.3	0.004	0.016	b, c
	2-Monostearin	GC-MS	24.66	129.1	0.008	0.026	b
	1-Monostearin	GC-MS	24.92	399.4	<0.001	<0.001	b, c
Glycerophospholipids	LPE 18:2	LC-MS (-)	9.10	476.2781	0.001	0.007	b, c
	LPE 20:4	LC-MS (-)	9.12	500.2782	<0.001	0.002	b, c
	LPE 16:0	LC-MS (-)	9.42	452.2785	<0.001	0.001	b, c
	LPE 18:1	LC-MS (-)	9.68	478.2933	0.001	0.004	b, c
	LPC 18:2	LC-MS (-)	9.21	564.3311	0.001	0.007	b, c
	LPC 20:4	LC-MS (-)	9.22	588.3310	<0.001	0.001	b, c
	LPC 16:0	LC-MS (-)	9.40	540.3312	<0.001	0.004	b, c
	LPC 20:3	LC-MS (+)	9.43	546.3567	<0.001	0.009	b
	LPC 18:1	LC-MS (-)	9.83	566.3465	0.006	0.019	c
Imidazopyrimidines	Urate	LC-MS (-)	0.94	167.0211	0.016	0.028	b, c
	Hypoxanthine	LC-MS (-)	4.49	271.0702	0.011	0.021	b, c
Non-metal oxoanions	Phosphoric acid	GC-MS	9.90	299.1	<0.001	0.001	c
Steroids and derivatives	TRH	LC-MS (-)	8.31	361.1635	<0.001	0.002	b, c
Phenols	Octopamine	GC-MS	12.10	174.1	<0.001	<0.001	b, c

ANOVA, analysis of variance; EVs, extracellular vesicles; GC-MS, gas chromatography coupled to mass spectrometry; Hex, hexose; K-W; Kruskall-Wallis ANOVA by ranks; LC-MS, liquid chromatography coupled to electrospray ionization mass spectrometry; LPC, lysophosphatidylcholine; LPE, lysophosphatidylethanolamine; MZ, mass-to-charge-ratio; p, probability value; PC, phosphatidylcholine; q, FDR adjusted p-value; RT, retention time; TG, triglycerides; TRH, thyrotropin

releasing hormone. Tukey's Honestly Significant Difference (HSD) or Dunn's multiple post-hoc comparisons test was used after ANOVA or Kruskal-Wallis ANOVA by ranks, to determine which specific group means differed: [a] HC vs. MDD, [b] HC vs. TRD, [c] MDD vs. TRD

Table S2. Differential metabolites detected by GC-MS and LC-MS analysis in plasma EVs of healthy controls (HC), patients with major depressive disorder (MDD) or with diagnosed treatment-resistant depression (TRD).

Class	Compound	HC vs. MDD				HC vs. TRD				MDD vs. TRD			
		p	VIP	q	FC	p	VIP	q	FC	p	VIP	q	FC
Benzene and substituted derivatives	2-Hydroxyphenylacetic acid	**0.005**	1.06	0.020	0.14	**0.004**	1.35	0.025	0.34	**0.001**	1.00	0.005	2.50
	3-Hydroxyanthranilic acid	0.023	1.01	NS	0.46	**<0.001**	1.19	0.002	0.20	**<0.001**	1.19	0.002	0.44
Carboxylic acids and derivatives	Propionic acid		NS		1.07	**0.004**	1.47	0.025	0.80	**0.004**	1.21	0.037	0.79
	Leucin	0.031	1.33	NS	1.09		NS		0.87		NS		0.79
	Sarcosine	0.040	1.16	NS	1.29		NS		1.19		NS		0.92
	Phenylalanine	0.036	1.32	NS	0.68		NS		1.27	0.017	1.27	NS	1.86
	Tryptophan		NS		0.76		NS		1.32	**0.002**	1.47	0.034	1.73
			NS		0.20	**0.001**	1.18	0.003	0.38	**0.001**	1.47	0.003	1.86
	Tryptophan betaine	0.037	1.12	NS	0.37		NS		0.70		NS		1.89
	Isoleucylglutamate		NS		0.76	0.039	1.52	NS	5.91	**0.005**	2.05	0.042	7.82
	Phenylacetylglutamine		NS		0.32	**0.012**	1.00	0.046	0.49	**0.019**	1.01	0.038	1.54
Fatty Acyls	Hexanoic acid		NS		0.90		NS		1.25	**0.002**	1.17	0.007	1.39
	Myristic acid		NS		0.97	**<0.001**	1.49	**<0.001**	1.97	**<0.001**	1.31	**<0.001**	1.65
	Arachidonic acid		NS		1.18	**<0.001**	1.62	**<0.001**	2.46	**<0.001**	1.48	**<0.001**	2.09
	Eicosapentaenoic acid		NS		1.22	**<0.001**	1.25	**<0.001**	2.43	**<0.001**	1.28	**<0.001**	2.00
	Butyric acid		NS		0.94	0.017	1.22	NS	0.70	**0.004**	1.03	0.037	0.75
	Oxononanoic acid		NS		1.09	**0.006**	1.21	0.028	1.16	**0.026**	1.06	0.047	1.06
	Leucic acid		NS		0.91	**0.003**	1.26	0.024	1.21	**0.029**	1.01	0.049	1.33
Glycerolipids	2-Monopalmitin		NS		1.22	**0.001**	1.23	0.002	1.76	**0.002**	1.08	0.005	1.44
	1-Monopalmitin		NS		1.23	**<0.001**	1.39	**<0.001**	2.50	**<0.001**	1.24	**<0.001**	2.02
	2-Monostearin		NS		1.13	**<0.001**	1.12	**<0.001**	1.87	**<0.001**	1.16	**<0.001**	1.68
	1-Monostearin		NS		1.09	**<0.001**	1.59	**<0.001**	2.72	**<0.001**	1.62	**<0.001**	2.50
Glycerophospholipids	LPC 16:1		NS		1.19	0.027	1.13	NS	1.51		NS		1.27
	LPC 20:3		NS		1.27	**0.003**	1.12	0.024	1.62	**0.003**	1.21	0.034	1.27
	LPC 16:0		NS		1.38	**0.008**	1.24	0.031	2.08	**0.013**	1.09	0.028	1.51
	LPC 18:1		NS		0.93		NS		1.28	0.037	1.00	NS	1.26
		0.046	1.10	NS	0.53	0.034	1.22	NS	0.87	**0.003**	1.18	0.010	1.63
	LPC 18:2		NS		0.82		NS		1.10	0.039	1.03	NS	1.35
		0.046	1.18		1.22	0.041	1.21	NS	1.99	**0.004**	1.25	0.011	1.63
	LPC 20:4		NS		1.29	0.032	1.15	NS	1.82	**0.003**	1.19	0.010	1.41
	LPE 16:0	**0.012**	1.44	0.046	0.81	**<0.001**	1.54	0.003	1.78	**0.001**	1.33	0.003	2.20
	LPE 18:1		NS		0.70	**0.005**	1.20	0.025	1.39	**0.001**	1.19	0.004	2.00
	LPE 18:2		NS		0.55	**0.004**	1.35	0.025	0.90	**0.001**	1.23	0.005	1.62

	LPE 20:4	NS	1.44	**0.001**	1.30	0.008	2.79	**0.003**	1.19	0.010	1.94
Organooxygen compd.	Galactose	NS	1.03	**0.005**	1.41	0.045	0.82	**0.011**	1.21	0.048	0.79
	Ribulose-5-phosphate	NS	0.97	**0.001**	1.00	0.003	0.87		NS		0.89
	Disaccharide(Hex-Hex)	NS	1.05	**0.005**	1.46	0.045	0.81	**0.003**	1.28	0.037	0.77
Imidazopyrimidines	Hypoxanthine	NS	0.40	**0.003**	1.08	0.009	0.70	**0.003**	1.08	0.009	1.74
	Urate	NS	1.50	**0.012**	1.03	0.045	2.14	**0.023**	1.08	0.044	1.42
Indolines	Indoline	NS	0.64		NS		1.20	**0.007**	1.27	0.042	1.88
Non-metal oxoanionic compd.	Phosphoric acid	NS	0.92		NS		1.12	**<0.001**	1.15	0.001	1.22
Steroids and derivatives	TRH	NS	0.16	**0.001**	1.72	0.014	0.10	**<0.001**	1.17	0.002	0.63
Phenols	Octopamine	NS	1.14		NS		0.77	**<0.001**	1.09	**<0.001**	0.66
Hydroxy acids and derivatives	6-Hydroxycaproic acid	NS	0.92		NS		0.71	0.026	1.68	NS	0.77

EVs, extracellular vesicles; GC-MS, gas chromatography coupled to mass spectrometry; FC, fold change; Hex, hexose; LC-MS, liquid chromatography coupled to electrospray ionization mass spectrometry; LPC, lysophosphatidylcholine; LPE, lysophosphatidylethanolamine; MZ, mass-to-charege-ratio; NS, non-significant; p, probability value; PC, phosphatidylcholine; q, FDR adjusted p-value; RT, retention time; TG, triglycerides; TRH, thyrotropin releasing hormone; VIP, variable importance in the projection.

https://doi.org/10.3390/biom16040533
